# Attenuated IL-2 muteins leverage the TCR signal to enhance regulatory T cell homeostasis and response *in vivo*

**DOI:** 10.3389/fimmu.2023.1257652

**Published:** 2023-09-22

**Authors:** Shining Ma, Michelle So, Aazam Ghelani, Rohith Srivas, Anupama Sahoo, Robyn Hall, Wenjun Liu, Hao Wu, Sherman Yu, Shiping Lu, Elly Song, Taryn Cariaga, Marcus Soto, Hong Zhou, Chi-Ming Li, Ashutosh Chaudhry, Xin Luo, Sue J. Sohn

**Affiliations:** ^1^ Amgen Research, Amgen Inc., South San Francisco, CA, United States; ^2^ Amgen Research, Amgen Inc., Thousand Oaks, CA, United States

**Keywords:** regulatory T cell (Treg), interleukin-2 (IL-2), IL-2 therapy, cytokine, T cell receptor (TCR), *in vivo*, tolerance

## Abstract

Interleukin-2 (IL-2), along with T-cell receptor (TCR) signaling, are required to control regulatory T cell (Treg) homeostasis and function *in vivo*. Due to the heightened sensitivity to IL-2, Tregs retain the ability to respond to low-dose or attenuated forms of IL-2, as currently being developed for clinical use to treat inflammatory diseases. While attenuated IL-2 increases Treg selectivity, the question remains as to whether a weakened IL-2 signal sufficiently enhances Treg suppressive function(s) toward disease modification. To understand this question, we characterized the *in vivo* activity and transcriptomic profiles of two different attenuated IL-2 muteins in comparison with wildtype (WT) IL-2. Our study showed that, in addition to favoring Tregs, the attenuated muteins induced disproportionately robust effects on Treg activation and conversion to effector Treg (eTreg) phenotype. Our data furthermore suggested that Tregs activated by attenuated IL-2 muteins showed reduced dependence on TCR signal, at least in part due to the enhanced ability of IL-2 muteins to amplify the TCR signal *in vivo*. These results point to a new paradigm wherein IL-2 influences Tregs’ sensitivity to antigenic signal, and that the combination effect may be leveraged for therapeutic use of attenuated IL-2 muteins.

## Introduction

Regulatory T cells (Tregs) play a critical role in establishing and maintaining immune homeostasis. While representing a relatively small proportion of circulating and tissue resident T cells, Tregs can exert a dominant effect by suppressing activated T and B cells, inducing a tolerogenic phenotype in antigen presenting cells, mediating tissue repair, and controlling metabolic adaptation [reviewed in (1, 2)]. These effects limit pathogenic inflammatory responses and restore homeostatic balance in tissues. Insufficient numbers or functional defects that render Tregs unable to control activated immune cells underlie the pathogenesis of many autoimmune and inflammatory diseases. For example, genetic deficiency of forkhead box p3 (*FOXP3*), a key transcription factor involved in Treg differentiation and functional specification, leads to the systemic autoimmune disorder, immune dysregulation polyendocrinopathy enteropathy X-linked syndrome (IPEX) in humans (3) and *scurfy* in mice (4, 5). Other genetic models targeting pathways that impact Treg number or function similarly capitulate dysregulation of immune homeostasis [reviewed in (6)].

Tregs respond to a multitude of stimuli that are available systemically and in tissue-specific contexts. Among these, T-cell receptor (TCR) and interleukin-2 (IL-2) signals play critical roles in the regulation of Treg number, phenotype, and function *in vivo* (7, 8). Because thymically derived Tregs are selected on self-antigen-derived peptides presented by MHC II, many Tregs experience antigenic signals *in vivo* at homeostasis (9, 10). Additionally, Tregs possess the ability to respond to limiting amounts of IL-2, made available by a few conventional CD4 T cells reacting to their cognate antigens (11). Utilizing the enhanced sensitivity of Tregs to IL-2, low-dose IL-2 (12) and various modalities of attenuated IL-2 (13–15), currently in clinical development, are intended to preferentially boost Tregs’ ability to curb pathogenic inflammatory responses in disease.

Previously, we shared our analysis of a large panel of engineered human IL-2 muteins that represented a wide spectrum of affinity and activity (16). Primarily using *in vitro* assays, we showed that attenuated IL-2 muteins, as a class, increased Treg selectivity and induced biological responses downstream of STAT5 activation. *In vitro* Treg responses to IL-2 muteins largely correlated with STAT5 activation, an IL-2 receptor (IL2R)-proximal signaling event. Here, to expand our understanding of Treg response to IL-2, and to understand whether attenuated IL-2 muteins induced key activation responses required for Treg function *in vivo*, we investigated the effects of wildtype (WT) and attenuated IL-2 muteins on Treg expansion and activation using mouse models. We performed transcriptomic analysis on Tregs from mice treated with IL-2 muteins, in combination with MHC II blocking antibody to reduce TCR signal to explore the contribution of TCR signal to Treg response to IL-2. We characterized molecular signatures and signaling pathways of IL-2 response in Tregs and compared the response with attenuated IL-2 muteins, and further explored IL2-stimulated Treg gene signatures in various biological contexts.

## Materials and methods

### *In vitro* IL-2 activity assay for mouse splenic Tregs

IL-2 activity was determined by STAT5 activation (phospho-STAT5 (pSTAT) signal in a flow cytometry-based assay. Mouse splenocytes were treated with ACK buffer (155mM Ammonium Chloride +10mM Potassium Bicarbonate +0.25mM EDTA in H_2_0, pH7.4), to lyse RBC and plated in complete RPMI media (RPMI1640 containing 10% FBS, buffered with HEPES GlutaMax, Penicillin-streptomycin, beta-mercaptoethanol, sodium pyruvate, and non-essential amino acid). Following a brief equilibration at 37°C, pre-diluted human WT IL-2, H16R, V91K D20A M104V (3x), D20W (all Fc-fused human IL-2 were produced at Amgen), or recombinant human IL-2 (R&D, cat# 10453-IL) was added to the wells and incubated for 20 min at 37°C. Cells were pelleted at 4°C and fixed in 1x Foxp3 fix/perm buffer and stained for Foxp3 using a kit (eBioscience cat # 00-5523). After Foxp3 staining, cells were fixed in Perm Buffer III of Phosflow kit (BD #558050) and stained for phospho STAT5, followed by extensive washing in FACS staining buffer (BD #554657), and stained for cell surface markers per standard protocol. pSTAT5 median fluorescence intensity (MFI) values from the Treg-gated population (Foxp3^+^ CD4^+^ CD3^+^ T cells) were used to generate IL-2 dose-response curves, and non-linear regression analysis was performed to generate EC50 values GraphPad Prism (v9.02; GraphPad Software, Inc., San Diego, CA).

### *In vivo* Treg responses

Characterization of the *in vivo* Treg response and bulk RNA-Seq analysis were performed in 8-12 week-old C57BL/6 female mice. IL-2 was dosed at indicated doses by subcutaneous injection. When specified, isotype control (LTF-2, BioXcell #BE0090) or MHC II blocking (M5/114, BioXcell #BE0108) antibody was administered by intraperitoneal injection at 500 μg per dose on day -1 and day 0. Animals were sacrificed on day 4 or 5 for analysis.

All experimental studies were conducted under protocols approved by the Institutional Animal Care and Use Committee (IACUC) of Amgen. The Amgen IACUC protocol number for IL-2 administration of mice is 2022-1526 and that for psoriasis model is 2022-1531. Animals were housed at Association for Assessment and Accreditation of Laboratory Animal Care (AAALAC) International-accredited facilities (at Amgen) in ventilated micro-isolated housing on corncob bedding. Animals had *ad libitum* access to sterile pelleted food and reverse osmosis-purified water and were maintained on a 12:12 h light:dark cycle with access to environmental enrichment opportunities.

### Mouse PK/PD studies and analysis

Male WT C57L/6 (7-8 weeks old) were purchased from Jackson Laboratory (CA, USA). Twenty-five micrograms of Fc-IL-2 proteins were dosed subcutaneously over the shoulders. Blood specimens were collected at various times post-injection (0.083h, 1h, 6h, 24h, 48h, 96h, 168h, 240h, 336h and 504h), incubated at ambient temperature for approximately 20 min or until fully clotted and then centrifuged to separate out the serum. All serum specimens were stored at -70°C ( ± 10°C) until use in analytical assays. PK parameters were estimated from mean serum concentration-nominal time data by noncompartmental analysis using Excel addins PKSolver (17). Serum concentration-time profiles were generated using GraphPad Prism (v9.02; GraphPad Software, Inc., San Diego, CA).

*In vivo* pSTAT5 response in mouse splenocytes was measured using the Phospho (Tyr694)/total STAT5a,b assay whole cell lysate kit (Meso Scale Discovery, #15163D) per manufacturer’s instructions and read on a Sector Imager 6000 (Meso Scale Discovery).

### Tissue processing and flow cytometric assay

#### Blood

Blood was collected from live mice 3 days post IL-2 treatment into EDTA tubes (BD #0266933). Blood was surface stained with antibodies diluted in BD Brilliant staining buffer (BD #566349) for 30 minutes on ice. Stained blood was RBC lysed by treatment with ACK buffer, washed and stained with Live/Dead Fixable Near IR Stain (Invitrogen #L10119) before overnight fixation/permeabilization with 1x Fix/Perm buffer at 4°C (eBioscience #00-5523-00). Intracellular antibody staining was performed at room temperature in 50μl of 1x Permeabilization buffer for 45 minutes, then washed according to vendor protocol and resuspended in BSA staining buffer (BD #554657).

#### Splenocytes

Spleens were dissociated in C-tubes (Miltenyi #130-093-237) in 10ml of cold MACS buffer (PBS, pH 7.2, 2 mM EDTA, and 0.5% BSA) using gentleMACS Octo Dissociator (Miltenyi,#130-095-937, protocol: “m_spleen_01”) and passed through 70μm cell strainer (Corning #431751). Cells were pelleted, RBC lysed in ACK Lysis buffer, quenched, refiltered through 70μm strainer, then resuspended in MACS buffer.

#### Skin

Subcutaneous fat was scraped away, tissue was minced with scissors, then incubated in 2ml of digest medium consisting of RPMI-1640, 5% FBS, 300μg/ml Liberase TM (Roche #05401119001), DNase I (Sigma #D4263) for 1.5hrs at 37°C while shaking at 100-200rpm. Digested fragments were disaggregated by pipetting, passed through 70μm strainer and resuspended in 50μl MACS buffer.

### Flow cytometry

Single cell suspensions of skin and splenocytes were surface stained with antibodies, washed with PBS, then Live/Dead stained, fixed and intracellularly stained as previously described (16). Skin samples were passed through 40μm filter tubes final time before data acquisition. Flow cytometry was performed on BD Symphony A3 Cytometer (BD, San Jose, CA, USA).

### Sample groups and Treg and Tcon cell purification for bulk RNA-seq analysis

Four different IL-2 treatment conditions (PBS, WT IL-2, H16R, and 3x mutein) within 2 antibody treatment groups (isotype and MHC II blocking antibodies) were evaluated with 4 mice in each group, resulting in a total of 32 Treg samples. CD25- Tcons from two mice from each group were included for comparison but were omitted from subsequent deep analysis. Treg and Tcon cells were purified from total splenocytes using a commercial mouse Treg isolation kit (Miltenyi # 130-091-041) following the manufacturer’s protocol. Individual mice were processed as separate samples. Tcons were collected as the flow-through fraction in the CD25 positive selection step. The bound fraction was eluted and collected as the Treg cells. Small aliquots were stained for CD3/CD4/CD25/Foxp3 to check for purity and indicated that most samples showed greater than 90% Foxp3^+^ staining post purification.

### TruSeq stranded mRNA library preparations

Sorted CD25- Tcon or Treg cell RNA was extracted by using Qiagen RNeasy mini kit (Qiagen #74106). The concentration and quality of the isolated RNA samples were assessed and determined on ThermoFisher NanoDrop 8000 spectrophotometer and Agilent 4200 TapeStation system, respectively, and had RNA integrity numbers at least 9.1. 200ng or 500ng RNA was used to prepare cDNA library by using a protocol modified from the Illumina TruSeq Stranded mRNA kit (Illumina #20020595). Briefly, after two rounds of polyA^+^ RNA selection and the step of fragmentation and priming at 94°C for 4 minutes, the fragmented RNA was reverse-transcribed to cDNA using SuperScript™ III (ThermoFisher Scientific Invitrogen™ #18080093) and RNase-out (ThermoFisher Scientific- Invitrogen™ #10777019) at 50°C for 1 hour. Sequentially, the products of the 1^st^ strand cDNA synthesis reaction were converted to double stranded cDNAs and subjected to end-repair, A-tailing, and adapter ligation by following up the commercial instruction. The constructed libraries were amplified and barcoded using the PCR program: a denaturation step at 98°C for 30 seconds, 15 cycles of 95 °C for 10 seconds, 60 °C for 30 second, 72°C for 30 seconds, and a 72 °C extension cycle for 5 minutes followed by a hold step at 4 °C. The amplified products were subjected to a clean-up step using AMPure XP bean (Beckman Coulter #63881) at the 0.85x ratio of beads to the PCR reaction volumes. After quantifying the clean-up products, the final barcoded libraries were sequenced to a minimum depth of 30 million paired-end reads at the 150nt read length on an Illumina Nextseq2000.

### RNA-Seq data analysis

RNA-Seq sequencing reads were aligned using OSA aligner (18) embedded in v10.0 of the Omicsoft Array Suite (Qiagen) to mouse genome version B38 based on gene model GENCODE v19. Gene level expression were quantified based on Omicsoft implementation of RSEM (19), and represented as normalized fragments per kilobase per million reads (FPKM). 2D Principal component analysis (PCA) plots were generated based on transformed, variance stabilized counts (using the variance-stabilizing transformation (VST) function in DESeq2) of all expressed genes for all samples or Treg samples to explore the distribution of samples across cell type and treatment conditions. Genes that were expressed at very low levels (counts per million mapped reads > 0.5 in less than 4 samples), genes that did not code for protein were filtered out before downstream differential expression analysis by DESeq2 (v1.30.1) with default parameters (20). Six pairwise comparisons were performed (WT vs PBS, H16R vs PBS, 3X vs PBS in Isotype and MHC II blockade conditions respectively). All differentially expressed genes (DEGs, FDR<0.1) were Z-score normalized versus PBS treatment, and clustered (hierarchical clustering, default parameters) and visualized in a heatmap using ComplexHeatmap (v.2.6.2) (21). Boxplots of example gene expression across conditions were generated by QIAGEN OmicSoft Studio (V11.7, Qiagen). UpSet plots were generated with UpSetR (v1.4.0) R package, to visualize the intersections of significantly up/down-regulated genes (FDR<0.1) in WT vs PBS, H16R vs PBS and 3X vs PBS in the isotype control antibody-treated group.

### WGCNA network analysis

We employed WGCNA (v1.71) R package to construct the weighted gene co-expression network (22, 23). Briefly, 19,662 genes with a total read count of more than fifty in all 32 Treg samples were kept for the analysis. The R function pickSoftThreshold was used to calculate the soft thresholding power and the optimal power was set as 5 to ensure scale-free topology fit index is above 0.9. A hierarchical clustering dendrogram of the topology overlap matrix (TOM) matrix was constructed with the parameter minModuleSize as 20 to identify gene co-expression modules. The module eigengenes (MEs) were defined as the first principal component expression of genes within a given module and calculated using the moduleEigengenes function in the R WGCNA package. It can be considered as a representative of the gene expression profiles within a module, which was further correlated with various sample traits by Pearson’s correlation.

### Pathway analysis

DEGs from different comparison group and WGCNA module genes were further analyzed by Reactome pathways (2016 version) using clusterProfiler (v3.18.1) (24) and ReactomePA (v1.34.0) R packages (25) or canonical pathways Ingenuity pathway analysis (IPA v01-20-04; Qiagen).

### Exploration of gene expression in public single cell RNA-Seq datasets

Lu et al. murine IL-2 treatment single cell RNA-Seq processed data were generated in house and previously published (26). Processed scRNA-Seq count matrix and metadata by Miragaia et al. (27) was obtained from https://figshare.com/projects/Treg_scRNA-seq/38864. Processed count data with cell classification from Reynolds et al. were downloaded from ArrayExpress (E-MTAB-8142). Processed count data were integrated with meta data provided by author, further filtered, and normalized to focus on Tregs with Seurat (v4.0.6) (28) R package. Gene expression across different Treg subgroups were visualized by DotPlot function by Seurat.

### *In vitro* Treg suppression assay

Tregs were isolated from total splenocytes using Treg purification kit according to manufacturer’s instructions (Miltenyi #130-091-041). CD8 T cells were isolated from naïve C57BL/6 spleens with EasySep purification kit according to manufacturer’s instructions (Stem Cell Technologies #19853). 1e5 CD8 T cells were co-cultured in 0.2ml complete RPMI media with 2.5e4 T cell activating Dynabeads (Gibco #11456D) and Treg at indicated ratios overnight at 37°C. Cells were washed, stained, acquired on BD Symphony cytometer and analyzed with Flowjo 10.5.3 (Treestar). Geometric mean fluorescence intensity (gMFI) of CD69 in the CD8 T-gated population was used as activation readout.

### Measurement of huIL-2 concentration in mouse serum

Quantification of Fc-IL2 proteins in mouse serum was performed using electro-chemiluminescent immunoassays on the Sector S 600 plate reader (Meso Scale Discovery) with biotinylated anti-human IL-2 antibody (R&D Systems, BAF202) as the capture reagent and ruthenylated mouse anti-human IgG Fc (Amgen clone 1.35.1 Mab) as the detection reagent. The interpolation of analyte serum concentrations from standard curves was performed in Watson LIMS™ Non-GLP Prod.

### Imiquimod psoriasis model

Six to eight week-old female BALB/c mice were purchased from Charles River Laboratory and maintained on a 12-hour light/dark cycle under specific pathogen-free conditions with access to food and water *ad libitum*. Protocols were approved by Institutional Animal Care and Use Committee of Amgen. Mice were administrated subcutaneously with 25μg IL-2 muteins or PBS a day before (day -1) imiquimod application. A topical dose of 62.5mg of Imiquimod cream (5%), containing 3.125mg active compound, (Aldara; AMA HEALTH CARE INCDBA BURTS PHARMACY) was applied daily on the shaved back skin and right ears for 5 consecutive days. All mice were weighed daily, and the percent body weight was calculated as the percent of the initial body weight. The ear thickness was measured daily with a caliper. Blood samples were collected on d2 (d3 post IL-2 muteins injection). Harvested tissues were processed for histopathological analysis or flow cytometry analysis.

## Results

### Attenuated IL-2 muteins induce a disproportionately robust Treg responses *in vivo*

To explore the effects of attenuated IL-2 signaling *in vivo*, we chose two human IL-2 muteins that exhibited attenuated activity compared to WT IL-2 in *in vitro* phospho-STAT5 (Tyr694 pSTAT5) assay. These IL-2 molecules were fused to effector function-less human IgG1 Fc, thus exhibiting reduced binding to Fc-gamma receptor and increased *in vivo* half-life. Both muteins demonstrated significantly differentiated levels of attenuation compared to WT IL-2. H16R showed approximately a 20-fold shift in EC50 compared to WT IL-2 in human Tregs (1.168 nM vs. 0.057 nM). D20A V91K M104V (hereon referred to as 3x mutein) exhibited approximately 84-fold reduction in activity by EC50 (4.763 nM vs. 0.057 nM) and significantly reduced Emax compared to both WT IL-2 and H16R and is thus considered a far-attenuated mutein. D20W served as a negative control because it binds to IL2RA weakly but does not induce significant pSTAT5 response *in vitro* (16).

**Figure 1:**
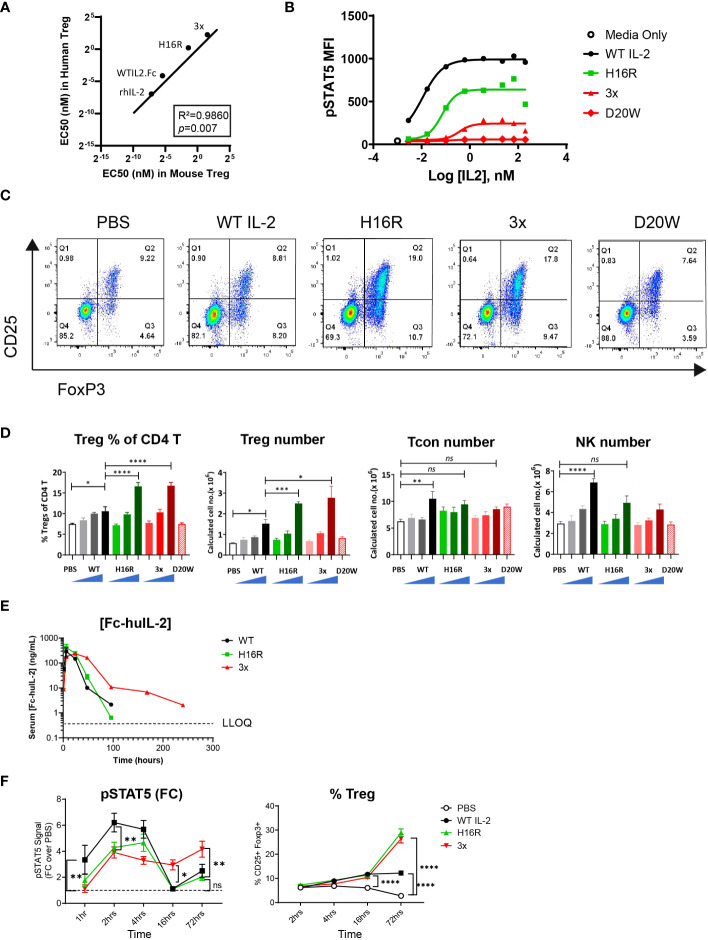
Robust and selective Treg expansion induced by attenuated IL-2 muteins *in vivo*. **(A)** Correlation analysis of the activity of human IL-2 molecules in human versus murine Tregs. The averaged EC50 values were determined in an *in vitro* STAT5 phosphorylation (pSTAT5) assay. The human EC50 values were from our previously published dataset and the mouse EC50 values are summarized in **Table 1**. The line indicates a hypothetical linear correlation with a slope=1. rhIL-2 (recombinant human IL-2), WT IL-2 (WT IL2-Fc fusion), H16R (human IL-2 mutein with H16R substitution), 3x (human IL-2 mutein with D20A, V91K, and M104V substitutions). The *R^2^* and *p* values (determined by two-tailed test) are shown. **(B)** Representative dose response curves for the human IL-2 in murine Tregs in the pSTAT5 assay. The pSTAT5 mean fluorescence intensity (MFI) in Treg population, gated as CD25^+^Foxp3^+^ CD4 T, in total splenocytes is shown against IL-2 concentration. **(C)** *In vivo* activity of human IL-2 in mice as shown by representative FACS plots. Tregs are captured as the CD25^+^ Foxp3^+^ cells amongst CD4 T cells, with percentages shown in each gate. **(D)** Summary of the Treg, Tcon, and NK cell response data. The percentages of Tregs as shown in **(C)** and the calculated total numbers of Treg, Tcon (Foxp3- CD4 T), and NK (CD3- CD49b+) cells from all treatment groups are summarized. D20W was administered at only the highest dose (25 μg). The bars represent the mean of the group and the error bars indicate standard error of mean (SEM). **(E)** Serum concentrations of WT IL-2, H16R, and 3x mutein at indicated time points post dosing. The mean of the group ± SEM (*n=3*) are shown. Concentrations that were below the lower limit of quantification (LLOQ) were omitted. **(F)** pSTAT5 signal as the fold change over control (PBS treated group at each time point) and the percentages of CD25^+^ Foxp3^+^ Tregs in CD4 splenic T cells at various time points post dosing are summarized. The mean of the group ± SEM (*n=5*) are shown. Two-way ANOVA with Tukey’s multiple comparisons test, *p < 0.05, **p < 0.01, ***p < 0.001, ****p < 0.0001, *ns*=not significant.

Using the STAT5 activation assay, we compared the rank order of activity of these human IL-2 molecules in human and mouse Tregs. As shown in **Figure 1A**, the EC50 values for human WT IL-2, the muteins, and recombinant IL-2 showed a strong correlation (*R*^2 ^= 0.9860) between human and mouse Tregs (**Table 1**). Additionally, the Emax values of the IL-2 muteins showed reduced maximal activities compare to WT IL-2 in mouse Tregs (**Figure 1B**), as previously observed in human Tregs. Of note, the activity of 3x mutein was less than 30% of WT IL-2 by Emax. D20W did not induce significant activity in mouse Tregs, similar to what was observed in human Tregs.

**Table 1:** Summary of the human IL-2 mutein EC50 values in human and mouse Treg cells.

	Average EC50, nM					
	WT IL-2	H16R	D20W	3x	rhuIL2	rmuIL2
Human Treg (published)	0.057	1.168	>10	4.763	0.008	ND
Mouse Treg (n=4)	0.023	0.378	Flat	2.916	0.007	0.005

Average EC50 values (in nM) from our previously published dataset (Ghelani et al., 2020) are shown for the human Tregs, and EC50 values for mouse Tregs were determined from the pSTAT5 assay performed with mouse splenocytes. Tregs were gated as CD25+Foxp3+ CD4 T cells and pSTAT5 MFI was used to generate IL-2 dose response curves. WT IL-2, WT IL-2.Fc; rhuIL2, recombinant human IL-2; rmuIL2, recombinant murine IL-2. D20W generated flat curves in the mouse cell assay so no EC50 values were generated. ND, not determined. Shown are the averaged values from 4 independent experiments.

To characterize the effect of WT and attenuated IL-2 *in vivo*, mice were given a single dose of 1, 5, or 25 μg of WT IL-2, H16R, or 3x mutein. The inactive D20W mutein was given only at the 25 μg dose. On day 4, spleens were harvested and analyzed by flow cytometry. As shown in **Figure 1C**, the percentages of CD25^+^ Foxp3^+^ CD4 T cells representing Tregs increased in mice treated with IL-2, particularly with H16R or 3x IL-2 mutein. Both the percentages of Tregs within the CD4 T cell compartment and total Treg numbers increased in a dose-dependent manner in response to WT IL-2, H16R, and 3x muteins (**Figure 1D**). D20W did not induce significant response. Interestingly, the maximal Treg response generated by WT IL-2 and the muteins did not correspond to the relative rank order of activity in the *in vitro* STAT5 activation assays. Both attenuated muteins robustly increased Treg number and representation to a significantly greater extent compared to that induced by WT IL-2. In contrast to Tregs, other cell types including Tcon (Foxp3^-^ CD4 T) and NK cells showed responses that were commensurate with the *in vitro* activity of these molecules. WT IL-2 significantly increased the total Tcon and NK cell numbers while H16R and 3x did not (**Figure 1D**). The combination of the heightened Treg and reduced non-Treg responses confirmed that attenuated IL-2 muteins increased Treg selectivity *in vivo*. Additionally, and perhaps more surprisingly, we observed that the attenuated muteins demonstrated a disproportionately robust activity on Tregs *in vivo*, incongruent with the level of activity measured *in vitro*. The fact that the 3x mutein, which is a far-attenuated mutein, enhanced Treg expansion as well as WT IL-2 was unexpected.

It has been proposed that attenuated IL-2 selectively enhances Treg response due to the prolonged half-life and reduced binding to non-Tregs *in vivo* (29). We performed a mouse pharmacokinetic (PK) study and measured the serum Fc-IL-2 concentrations at various time points after dosing. We observed that the kinetics of WT and H16R were similar, as their concentrations dropped to undetectable levels beyond 96 hours (**Figure 1E**). In contrast, the serum levels of 3x mutein remained sustained and was detectable up to 240 hours. Compared to the reported half-life of Proleukin [0.74h, (30)], the half-lives of the Fc-fused WT, H16R, and 3x mutein were much longer, at 12.17, 8.43, and 29.3 hours respectively (**Table 2**). Additional PK parameters of Fc-IL-2 were generally consistent with the notion that IL-2 with reduced affinity to the receptor remained in circulation longer, although this effect was much more pronounced for 3x mutein compared to H16R.

**Table 2:** Summary of the mouse *in vivo* PK measurements of Fc-huIL-2.

	WT IL-2	H16R	3x
AUC (ng*Hours/mL)	6219.82	9424.18	12640.89
Cmax (ng/mL)	292	412	237
Cl/F obs(mL/Hours)	4	2.56	1.97
T1/2 (Hours)	12.17	8.43	29.3

Calculated area under the curve (AUC) of the measured serum Fc.IL-2 concentration, the maximal concentration achieved (Cmax), apparent clearance (Cl/F obs)), and half-life (T1/2, time to reach 50% of the Cmax) for WT IL-2, H16R, and 3x mutein were determined in a mouse PK study and summarized. These values were calculated from the mean concentration values as described in the methods section.

We evaluated the *in vivo* pharmacodynamic (PD) activity of IL-2 by measuring the pSTAT5 signal and Treg expansion in splenocytes. Our data showed that the peak of the pSTAT5 signal was seen between 2 and 4 hours for all three IL-2 molecules, with WT IL-2 reaching the highest peak signal intensity compared to H16R or 3x mutein (**Figures 1F**, **S1**). Interestingly, 3x mutein uniquely maintained elevated pSTAT5 signals through much later time points, extending to 16 hours and beyond. The percentages of Tregs also increased over time, with delayed kinetics compared to the pSTAT5 response. The increase was seen as early as 16 hours post IL-2 dosing and further increased at 72 hours, at which point the two attenuated muteins showed greater Treg expansion compared to WT IL-2. These data showed a correlation between 3x mutein’s ability to generate a sustained IL-2 signal and Treg response *in vivo*. However, the PK and PD profiles of WT IL-2 and H16R were similar; thus it is unclear whether the superior activity of H16R in Treg expansion can be explained solely by its PK and PD profile.

### TCR signal is required for IL-2-driven Treg expansion but not activation

**Figure 2:**
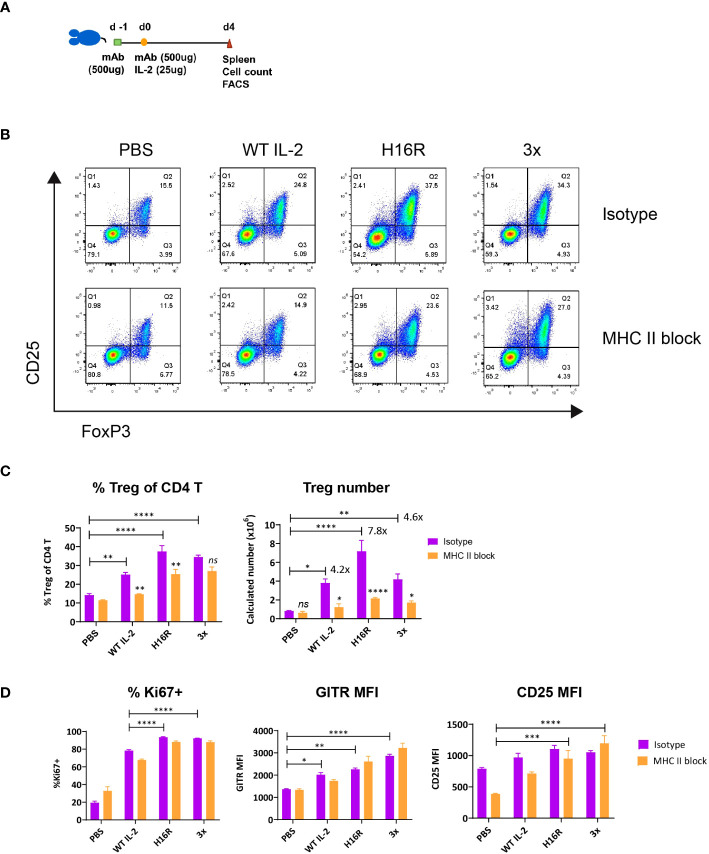
The impact of blocking the TCR signal on IL2-mediated Treg expansion and activation. **(A)** Study design. C57BL/6 mice were treated twice with isotype control or MHC class II blocking antibody (500μg per dose by i.p. injection) on day -1 and d0 and dosed with IL-2 on d0 (25μg by s.c. injection). Spleens were harvested on d4 post IL-2 dosing and analyzed. **(B)** Representative flow profiles show the Foxp3^+^ CD25^+^ CD4 Treg response to IL-2 with (bottom row) or without (top row) the MHC II blockade treatment. **(C)** Treg expansion in response to IL-2 with or without the MHC II blockade *in vivo*. Percentages of Treg in CD4 T as defined in **(B)** and calculated splenic Treg cell number are summarized for the groups treated with PBS, WT IL-2, H16R, or 3x mutein with pre-treatment with isotype (purple bars) or MHC II blocking (orange bars) antibody. The fold increase in Treg cell number, calculated by taking the ratio of Treg number in IL2-treated group over PBS-treated group is indicated for the isotype antibody-treated groups only. **(D)** Treg activation in response to IL-2 with or without the MHC II blockade *in vivo*. Percent Ki67^+^ and GITR MFI of Foxp3^+^ CD25^+^ Tregs and CD25 MFI of Foxp3^+^ CD4 T cells are summarized. **(C, D)** Shown are the mean values for the group (*n*=3) and the error bars indicate the SEM values. Two-way ANOVA with Tukey’s multiple comparisons test, *p < 0.05, **p < 0.01, ***p < 0.001, ****p < 0.0001. Data are representative of three independent studies.

**Figure 3:**
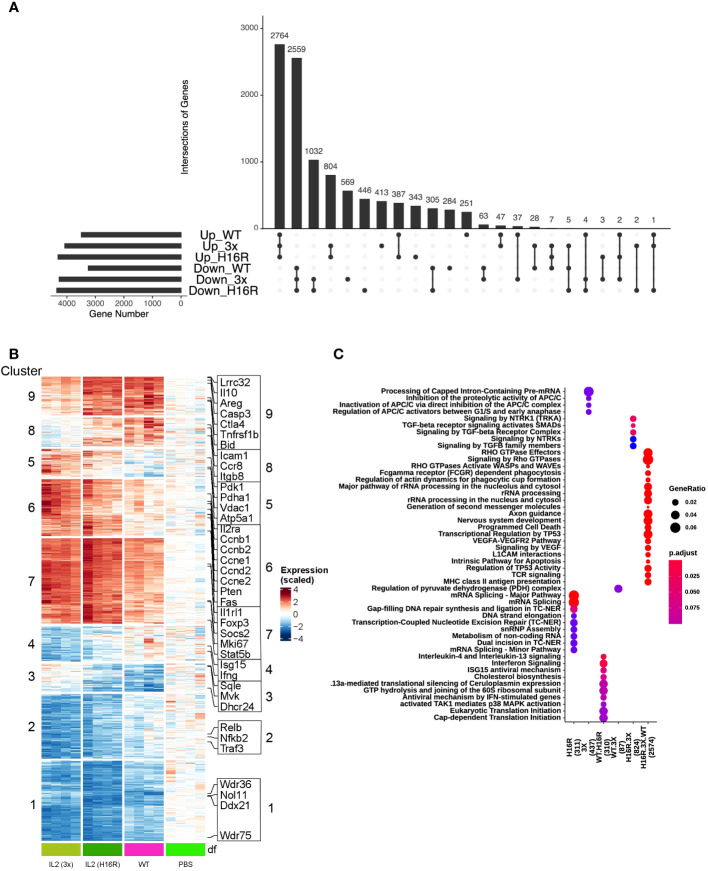
Characterization of Treg transcriptional response to WT IL-2 and attenuated muteins *in vivo*. **(A)** UpSet plot visualizing intersections of significantly up/down-regulated differentially expressed genes (FDR<0.1) in WT vs PBS, H16R vs PBS and 3x vs PBS in the isotype control antibody-treated group. The numbers above the bars indicate the number of intersected genes. The total gene number for each category is shown on the left. **(B)** Heatmap with hierarchical clustering showing expression of all DEGs in **(A)** across treatment conditions. **(C)** Comparison of top representative Reactome pathways among the various groups of DEGs as in **(A)** top middle panel (for all DEGs). Only top 5 significantly enriched Reactome terms (adjusted *p* value<0.25) are shown. The color of dots represents increasing significance of the enriched pathways from blue to red and the size of the dot reflects the GeneRatio (Proportion of DEGs in a specific comparison group that associated with the given Reactome Term). Number of DEGs analyzed in the gene sets are in parentheses.

Treg responses to IL-2 can be influenced by environmental signals beyond IL-2 availability. These include signals generated by the TCR (8), costimulatory receptors [e.g., CD28 (31), ICOS (32)], tumor necrosis factor receptor (TNFR) superfamily members [e.g., 4-1BB (33), OX40 (34), GITR (35)], cytokines [e.g., IL-33 (36), TGF-β (37)], and hormones such as leptin (38). We hypothesized that weak IL-2 muteins synergized with one or more of these signals *in vivo* to induce robust Treg response, rather than acting solely through STAT5 activation immediately downstream of the IL2R complex. Since TCR signal is an important driver of Treg responses, we focused on evaluating the contribution of TCR signal to the IL2-dependent Treg response. To this end, mice were pretreated with isotype control or MHC II-blocking antibody, followed by administration of a single dose of IL-2 (**Figure 2A**). IL-2 treatment increased the representation and the number of Tregs among CD4 T cells when the mice were pre-treated with the isotype control antibody, similar to the previous studies without any antibody treatment. In contrast, the Treg response was significantly diminished when mice were pretreated with MHC II blocking antibody (**Figure 2B**). By cell number, Treg expansion was greatest with the slightly attenuated mutein H16R, at nearly 8-fold increase over the PBS treated control (**Figure 3C**). The far-attenuated 3x mutein induced a similar Treg expansion response as WT IL-2. MHC II blockade dramatically reduced this response, indicating that the TCR signal is required for IL-2-driven Treg expansion *in vivo*. We also evaluated Treg activation response to IL-2 by assessing the expression of several known activation markers including Ki67, Gitr and Cd25. Both H16R and 3x muteins robustly induced expression of Treg activation markers, and the far-attenuated 3x mutein exerted an even stronger effect than WT IL-2 (**Figure 2D**). Furthermore, and in contrast to the Treg cell number, blocking the TCR signal had little to no effect. Tcons did not upregulate Ki67 or Gitr *in vivo* in response to any of the IL-2 molecules and the MHC II blockade had no impact (**Figure S2**), indicating that IL-2 selectively activated Tregs. These data clearly demonstrated the distinct requirement of TCR signal in IL2-dependent Treg expansion versus activation and suggested that the attenuated muteins could activate Tregs even when the TCR signal was compromised.

### Attenuated IL-2 muteins modify the core IL-2 gene signature with biological implications

We sought to further understand how attenuated IL-2 muteins generated strong Treg activation responses *in vivo* by performing transcriptomic analyses. Applying the same study design to treat animals with isotype control or MHC II blocking antibody followed by IL-2 (**Figure 2A**), purified Tregs on day 4 were analyzed by bulk RNA-seq analysis. We also included Tcons (CD25- CD4 T cells) from two of the four mice from each treatment group as comparison. Principal component analysis (PCA) revealed a clear separation between the Treg and Tcon samples, confirming successful cell purification and expected distinct transcriptional programs of Tregs and Tcons (**Figure S3**). PCA of Treg samples revealed that the maximum variation was driven by treatment conditions; all IL-2 treatment conditions were clearly separated from PBS control, with the two IL-2 muteins further clustering apart from the WT IL-2 treatment. We observed further data separation based on antibody treatment (isotype versus MHC II blocking), particularly amongst samples treated with IL-2 (WT and the two muteins). The PBS-treated samples showed no separation based on antibody treatment, suggesting that blocking TCR signal by itself had little impact in the absence of IL-2 treatment within the short timeframe (4 days) of this study. Taken together, these data suggest a prominent change in gene expression following treatment with IL-2, with impact from the TCR blockade, particularly in the IL2-treated samples.

We analyzed the transcriptional response induced by WT IL-2, H16R and 3x mutein in the isotype antibody-treated group and performed differential gene expression analysis comparing the changes observed under IL-2 treatment versus baseline (PBS treatment). We set low cutoff thresholds (FDR<0.1, no fold change cutoff) to broadly capture all changes that may be of significance in the biological context. Comparison of the differentially expressed genes (DEGs) across different treatment groups was visualized by the UpSet plot (**Figure 3A**), which showed that IL-2 treatment led to both increased and decreased gene expression. At an FDR < 0.1, we observed a total of 10356 differentially expressed genes (DEGs) between any IL-2 treatment versus PBS. The majority (52% or 5342 genes) of the DEGs overlap across all treatments, with 48% (2559 out of 5347 genes) in downregulated and 54% (2764 out of 5098 genes) in upregulated DEGs. However, we also observed significant changes linked only to the IL-2 mutein treatments, indicating a potentially unique transcriptional response associated with attenuated IL-2 muteins.

To map out the gene response patterns across treatments, we subjected the set of DEGs identified in **Figure 3A** to unbiased hierarchical clustering. We identified a total 9 different clusters as indicated in a heatmap in **Figure 3B**. Clusters 1 and 2 captured genes that were largely downregulated by all IL-2 treatments, with the attenuated muteins inducing a greater degree of downregulation compared to WT IL-2. Clusters 5 - 9 captured genes that were largely upregulated across all IL-2 treatments. Clusters 8 and 9 showed a pattern that correlated with the *in vitro* activity of the three IL-2 molecules, i.e., ranking in the order of WT>H16R>3x by pSTAT5 response, while clusters 6 and 7 showed an opposite trend. Clusters 3 - 5 captured small numbers of genes that appeared to show conflicting trends amongst the different IL-2 species.

Surveying for a few exemplary genes that are commonly identified as IL-2 response genes in Tregs showed that they were scattered across different clusters (**Figure S4** and **Supplementary data file S1**). We found many genes canonically associated with Treg function including *Lrrc*32 (*Garp*), *Il10*, *Ctla4*, and Amphiregulin (*Areg*) in cluster 9, and these genes showed a stronger response to WT and H16R than to 3x. *Il2Rra* (*Cd25*) was found in cluster 6 along with many genes involved in cell cycle regulation (e.g., *Ccnb1*, *Ccnb2*, *Ccnd2*), and these genes were induced to a greater extent by H16R and 3x mutein than WT IL-2. The fact that cell cycle regulators were expressed highly by the attenuated muteins was consistent with the greater expansion of Tregs associated with the muteins. Others such as *Ki67*, *Il1rl1* (*St2*), *Foxp3*, *Socs2*, and *Stat5b*, that are known to contribute to IL-2 and/or Treg response pathways were found to be elevated by all three IL-2 species in cluster 7. Thus, attenuated IL-2 muteins can generate a canonical IL-2 activation response in Tregs, although with some differences compared to the WT IL-2 response.

To better understand the differences between WT IL-2 and mutein-generated responses in Tregs, we performed enriched pathway analysis based on Reactome 2016 (25), which suggested that individual clusters were associated with multiple pathways with overlapping biological implications (**Figure S4**). IL-2 treatment prominently increased expression of genes (**Figure S4**) that broadly regulate immune responses including some key Treg-associated genes as mentioned above (cluster 9), genes in the chemokine and cell adhesion receptor pathway (cluster 8, *Ccr8*, *Icam1*, *Itgb8*), and metabolic pathways such as pyruvate dehydrogenase complex (cluster 5, *Pdk1*, *Pdha1*, *Atp5a1*, *Vdac1*), consistent with the known role of IL-2 in Treg activation, migration, and metabolic reprogramming. The attenuated IL-2 muteins enhanced cell cycle (cluster 6) and axon guidance/Rho GTPase pathway (cluster 7, *Cdc42*, *Pak2*, *Nck1*, *Nck2*) responses, consistent with the robust Treg responses observed in mice treated with H16R or 3x mutein. Pathways involved in cholesterol biosynthesis (cluster 3, *Mvk*, *Dhcr24*, *Sqle*), TNFRSF-mediated non-canonical NF-kB pathway (cluster 2, *Nfkb2*, *Relb*, *Traf3*), and ribosomal RNA (rRNA) modification pathway (cluster 1, *Ddx21*, *Nol11*, *Wdr36*, *Wdr75*) were repressed in response to IL-2. Subtle differences between WT IL-2 and attenuated muteins were captured in cluster 4 which included several interferon signaling pathway genes (e.g., *Irf7*, *Isg15*, *Ifit1*), with greater degree of repression associated with the muteins, and an opposite trend in cluster 3 (the cholesterol biosynthesis pathway) where WT IL-2 led to a greater degree of repression.

Shared gene responses amongst the different IL-2 molecules are summarized in a dot plot in **Figure 3C**. Genes regulated by all three IL-2 molecules (2574 genes) are associated with RHO GTPases, phagocytosis, programmed cell death, and TCR signaling pathways, which point to fundamental effects of IL-2 on Tregs. Altered genes shared by WT and H16R (310 genes) are enriched for interferon signaling and cholesterol biosynthesis, while WT and 3x mutein shared only a small number of genes (87 genes) associated with pyruvate metabolism. Genes uniquely altered by both muteins (824 genes) are enriched for TGF-beta and NTRK signaling, providing additional insights into how IL-2 muteins may enhance Treg function, considering the role of TGF-beta signaling in promoting Treg function and peripheral Treg cell differentiation. This strengthens the notion that IL-2 (or specifically, the attenuated muteins) enforces Treg function and phenotype by feed-forward mechanisms. Furthermore, 3x mutein uniquely regulated genes (437 genes) highlighting additional cell cycle-related pathways, whereas H16R uniquely regulated genes (311 genes) involved in mRNA processing, DNA repair and snRNP assembly. These results suggest that the attenuated IL-2 muteins modulated shared and unique biological processes in Tregs compared to WT IL-2.

### Attenuated IL-2 muteins highlight Treg gene signatures that are co-regulated by the TCR signal

**Figure 4:**
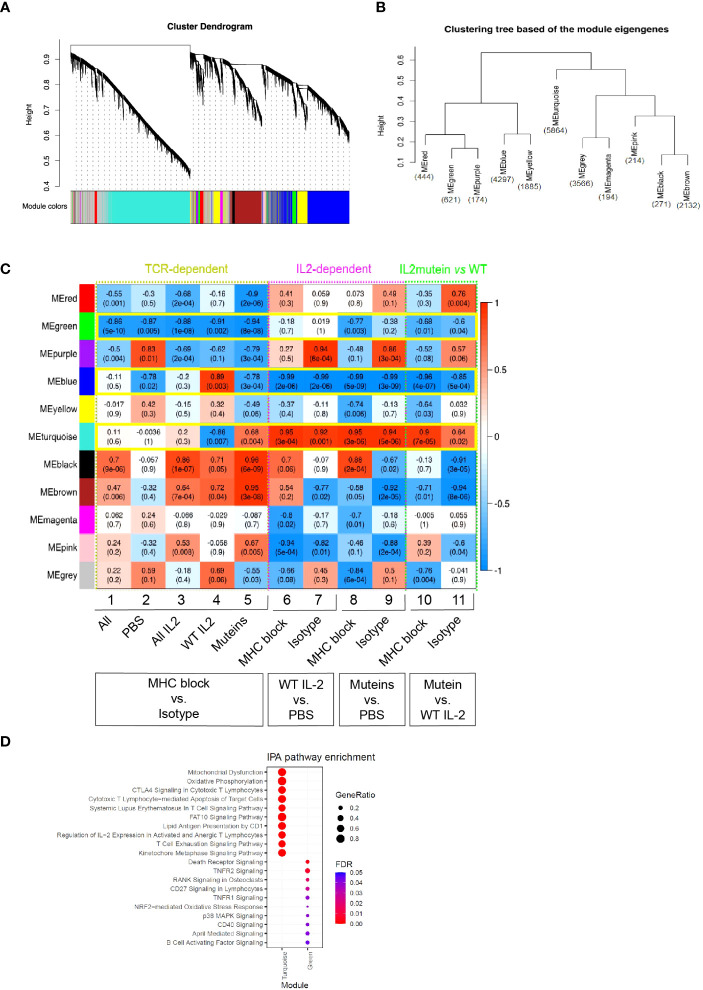
Weighted correlation network analysis (WGCNA) and identification of modules of genes that are co-regulated in response to IL-2 treatment and MHC II blockade. **(A)** Hierarchical clustering dendrograms of co-expressed genes in modules in 32 Treg samples, with dissimilarity based on topological overlap. Each leaf in the dendrogram represents one gene and the colored row underneath the dendrogram represents color-coded modules which contain a group of highly connected genes. A total of 11 modules was identified. **(B)** Clustering tree of module eigengenes which summarize the modules identified in **(A)**. Module eigengene is the first principal component of the corresponding module. The number of genes in each module was presented in blue. **(C)** Correlation heatmap of WGCNA module genes and traits (module-trait relationship). Each row represents a module, and the columns correspond to eleven traits, including TCR-dependent (MHC II block vs. Isotype comparison, columns 1-5), IL2-dependent (IL-2 vs. PBS comparison, columns 6-9) and IL-2 mutein vs WT traits (columns 10 and 11). Each cell contains the corresponding Pearson correlation coefficient and *p*-value, of which red represents positive correlation with the first phenotype in each column and blue represents negative correlation. The correlation increases as the color darkens. Modules demonstrating a consistent relationship with the trait of interest are highlighted in green boxes. **(D)** Dot plot summarizing the top 10 significantly enriched canonical pathways (adjusted *p* value<0.05) associated with turquoise and green modules identified using Ingenuity Pathway Analysis (IPA) (39). The color of dots represents increasing significance of the enriched pathways from blue to red and the size of the dot reflects the GeneRatio (Proportion of DEGs in a specific comparison group that associated with the given ingenuity canonical pathway).

The inclusion of MHC II blockade treatment showed that certain aspects of the Treg response to IL-2 were co-regulated by the TCR signaling while others were not. To investigate the functional gene regulatory network underlying the IL2-induced transcriptional responses and the crosstalk with TCR signaling, we performed weighted correlation network analysis (WGCNA) (22, 23) of gene expression and identified 11 co-expression modules of genes (**Figures 4A, B**). The relationship amongst the modules and the number of genes in each module are presented in a dendrogram representation, which highlighted that the turquoise module, containing the largest number of genes, separated out as a distinct module.

We performed correlation analysis to understand how individual modules related to various treatment conditions and summarized the result in a heatmap (**Figure 4C**). For example, columns 1-5 show how the modules associate with MHC II blockade vs. isotype treatment across different IL-2 treatment conditions. The green module showed a statistically significant negative correlation with MHC II blockade, and thus it represented a gene module that showed dependence on the TCR signal regardless of the IL-2 status.

Similar analysis was performed across columns 6-11 to characterize IL2-dependent gene responses, and the turquoise module emerged as a prominent module that showed consistently positive correlation with the IL-2 treatment compared with the PBS controls. The turquoise module showed sensitivity to TCR blockade with WT IL-2 treatment, while the attenuated muteins elicited exaggerated responses that were not diminished by TCR blockade (**Figures 4C**, **S5**). This pattern suggested that WT IL-2 and TCR signals synergistically induced this module but that attenuated muteins could stimulate response even when TCR signal was limited. In contrast, the blue module was suppressed by IL-2 treatment, with greater suppression by muteins compared to WT. TCR blockade prevented the WT IL2- but not the attenuated mutein-mediated response.

The red and purple modules also showed a trend of negative correlation with MHC class II blockade, with the attenuated muteins generating stronger effect compared to WT IL-2. These trends suggested that the attenuated muteins induced a distinct signal from WT IL-2, with an ability to amplify signals where TCR signal is limited or reduced.

To explore biological processes represented by these gene modules, we conducted pathway enrichment analysis using Ingenuity Pathway Analysis (IPA), focusing primarily on the green module as the Treg gene module regulated by the TCR signal, and the turquoise module as that regulated by the combination of the IL-2 and TCR signals, with amplified induction by the attenuated muteins.

As shown in **Figure 4D**, the genes in the turquoise module were significantly enriched in pathways related to cellular metabolism, including mitochondrial dysfunction (e.g., *Casp3*, *Gpx7*, *Prdx5*, *Sod2*) and oxidative phosphorylation (e.g., *Cyb5a*, *Ndufa11*, *Uqcrc1*). T cell signaling-related pathways were also highlighted as the CTLA4 signaling in cytotoxic T (e.g., *Akt1*, *Cd3d*, *Lck*, *Zap70*), SLE in T cell signaling pathway (e.g., *Foxp3*, *Icos*, *Rhog*, *Stat3*), regulation of IL-2 expression in activated and anergic T lymphocytes (*Calm1*, *Nfkb1*, *Smad3*, *Tgfb1*), T cell exhaustion signaling pathway (e.g., *Batf*, *Foxo1*, *Il10rb*, *Pdcd1*, *Prdm1*, *Tigit*) and PI3K/AKT signaling pathway (*Il1rl1*, *IL2ra*, *Itgb7*). These gene profiles and associated pathways suggest that IL-2 and TCR signals synergize to enhance Treg’s metabolic fitness and increase their function, and the attenuated IL-2 muteins further amplified these effects with reduced requirement for TCR signaling. The genes in the green module were significantly enriched in the TNFRSF-related signaling pathways as suggested by death receptor signaling (e.g., *Nfkbid*, *Rel*), RANK signaling in osteoclasts (e.g., *Map2k6*, *Traf6*), NRF2-mediated oxidative stress response (e.g., *Atf4*, *Sqstm1*) and p38 MAPK signaling pathways. Since the green module genes are expressed in a TCR-dependent manner, this suggested that the TCR signal is essential for engaging these pathways in Tregs.

**Figure 5:**
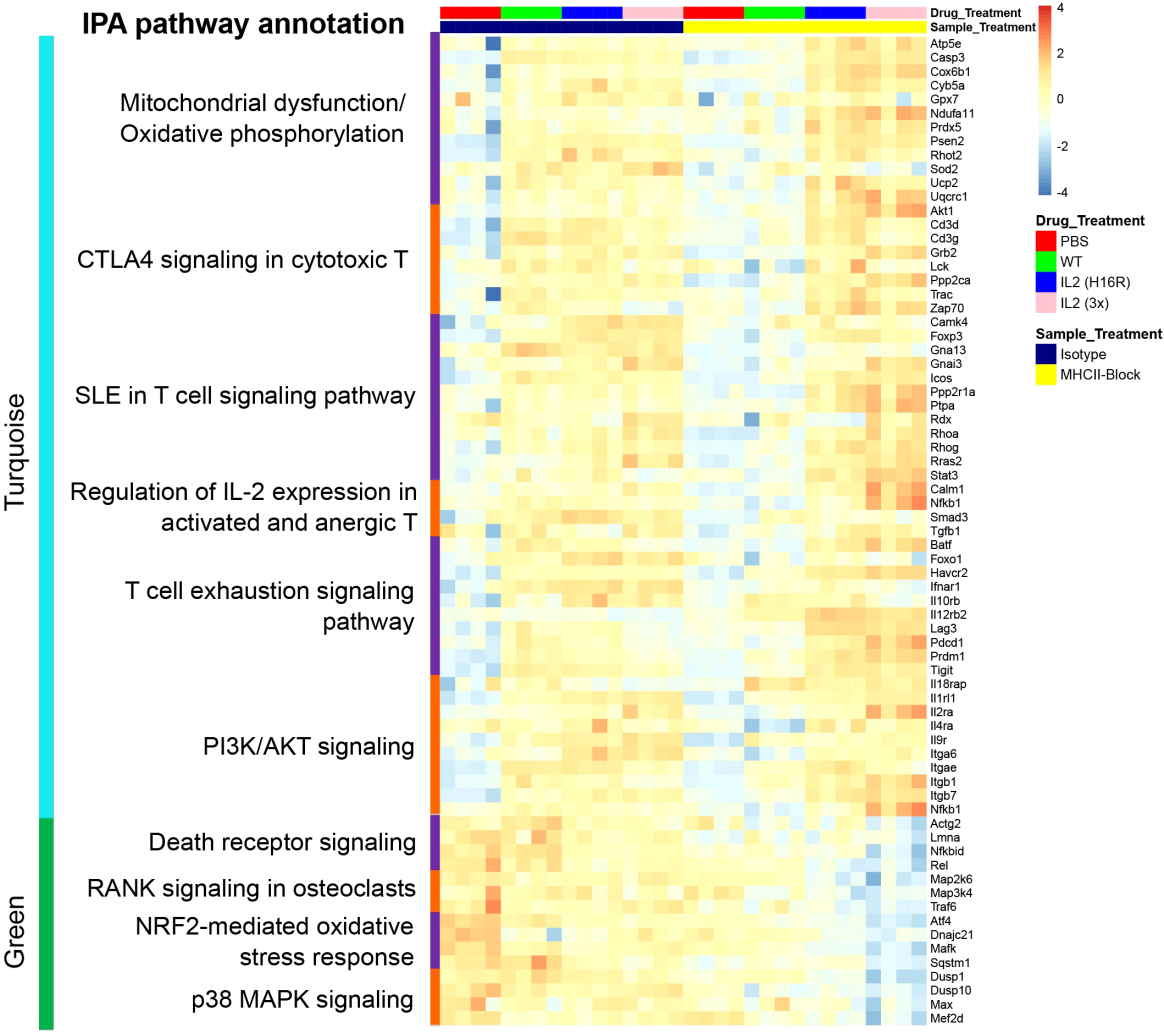
Heatmap summarizing the scaled normalized expression for individual genes of interest in the key pathways in the turquoise (top) and the green (bottom) modules across treatment conditions. Each column represents a Treg sample.

To illuminate biological pathways that are relevant to Treg states and function that underlie homeostatic and disease conditions, we curated a list of genes representing the pathways in the turquoise and green modules, which are summarized in a heatmap (**Figure 5**). These genes showed expression patterns that were consistent with the module response pattern as indicated by the module-trait analysis and were used as reference genes to further query pathway involvement in Treg responses *in vivo*.

### IL-2 treatment amplifies activated Treg gene signatures associated with non-lymphoid tissue Tregs

**Figure 6:**
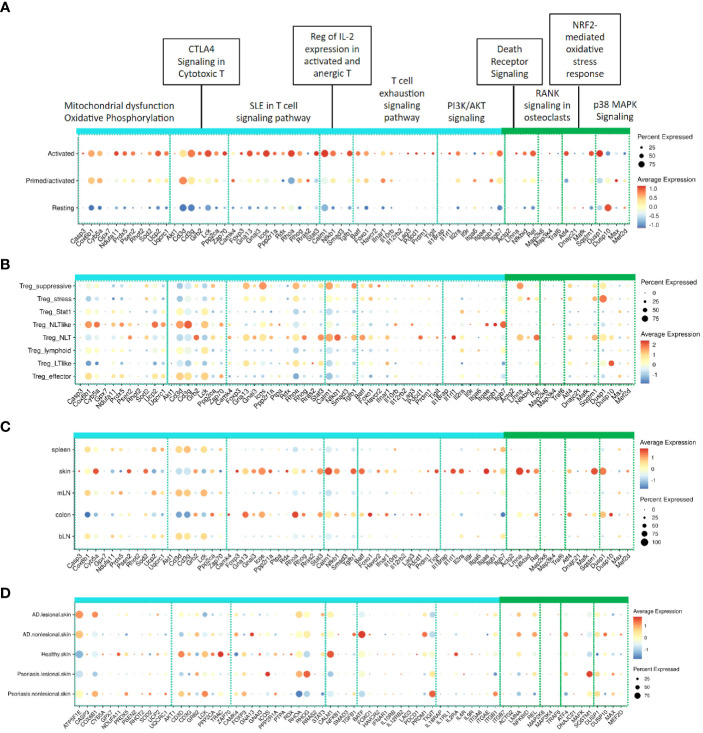
Expression of representative signature genes in the turquoise and green modules in related published mouse and human scRNA-Seq datasets. **(A)** Expression across activation states in mouse Tregs stimulated with muIL-2 as defined in Lu et al. (26); Expression across **(B)** Treg subpopulations and **(C)** Tregs found in different tissues as defined by Miragaia et al. (27); **(D)** Distribution across healthy tissue from healthy donor, non-lesional and lesional skin tissues from atopic dermatitis and psoriasis patients in Reynolds et al. (40), The color of dots indicates the scaled average expression in all cells in the corresponding group and the size of dots represents the percentage of the cells with at least one transcript detected.

We used a previously published single cell RNA seq (scRNA-Seq) dataset generated from mouse Tregs responding to a mouse IL-2 mutein (26) to confirm the relevance of our reference genes, which were generated from mouse Tregs responding to human IL-2 muteins. As shown in **Figure 6A**, these genes were largely enriched in the Treg population that had been annotated as “activated” by IL-2 compared with the “resting” Treg population. The transitional population annotated as “Primed/activated” showed intermediate expression. This analysis demonstrated that mouse Tregs responded to human IL-2 *in vivo* similarly as they did to murine IL-2, and the gene responses captured by the turquoise and green modules reflected the Treg activation state.

To deconvolute which Treg subsets responded to IL-2, we used a comprehensive mouse Treg dataset from a study published by Teichmann and colleagues (27), which had defined functional Treg subsets including Treg_stress, Treg_STAT1, Treg_NLT-like, Treg_effector (eTreg), and Treg_suppressive, which were proposed to represent the various stages of Treg adaptation during homeostatic circulation based on kinetic trajectory analysis. Additionally, it had mapped subpopulations of Tregs in lymphoid tissues (spleen, brachial lymph node (bLN) and mesenteric LN (mLN), collectively referred to as lymphoid tissue, or LT) and the mucosal barrier tissues (skin and colon, referred to as non-lymphoid tissue, or NLT). Querying this dataset for the expression of the turquoise and green module genes showed that these genes were preferentially enriched in Treg subsets that had been annotated as suppressive, NLT, NLT-like, and to a lesser extent, eTreg subpopulations (**Figure 6B**). Based on these results, we propose that IL-2 promotes Tregs to adapt aspects of the tissue-resident NLT phenotype and to take on functional traits of effector and suppressive Treg cells. Additionally, transcriptional signatures accompanying these phenotypic changes can be captured in lymphoid tissues like the spleen.

The turquoise and green module gene signatures were prominently enriched in Tregs from NLTs, such as the skin and the colon (**Figure 6C**). Among the turquoise module pathways, the SLE T cell signaling pathway, regulation of IL-2 expression, and PI3K/AKT signaling pathways (mostly represented by cytokine receptor and adhesion receptor genes) were highlighted, while the green module showed a prominent association with the death receptor signaling. Interestingly, the CTLA4 signaling in cytotoxic T pathway genes, including those proximally involved in TCR receptor signaling (e.g, *Cd3d*, *Cd3g*, *Lck*), showed an opposite trend and these genes appeared to be expressed at slightly higher levels in LT than in NLT Tregs. The mitochondrial dysfunction and oxidative phosphorylation pathway genes also tended to be expressed at higher levels in the LT Tregs. These results indicate alternative IL-2 response profiles between LT and NLT Tregs, which consequently may reflect differences in their sensitivity to TCR signaling, tissue tropism, and/or metabolic response to their environment.

One hypothesis around inflammatory diseases is that reduced number of Tregs and/or activation limit the suppression of inflammatory response in tissues. To evaluate Treg activation state in human diseases, we characterized the turquoise and green module Treg gene signatures in human inflammatory disease datasets. Because these gene modules showed a compelling association with skin-resident Tregs, we focused our analysis on two human skin inflammatory diseases, atopic dermatitis and psoriasis, using the dataset published by Teichmann, Watt, Haniffa and colleagues (40). We queried the turquoise and green module reference genes to determine whether Tregs displayed activation gene signatures in healthy versus inflamed tissues (**Figure 6D**). In this analysis, IL2-induced Treg activation genes were modestly represented across healthy, non-lesional or lesional tissues, with some that differed between healthy vs non/lesional skin. For example, the CTLA4 signaling in cytotoxic T pathway genes in the turquoise module were expressed robustly in healthy skin but appeared reduced in both lesional and non-lesional skin in AD and psoriasis. The genes in the green module were not expressed in Tregs in healthy skin, while a few (e.g., *Lmna*, *Rel*, *Atf4*, *Sqstm1*, *Dusp10*) appeared to be elevated in skin Tregs in disease patients. Overall, the inflamed skin Tregs did not express high levels of IL-2 activation signature genes, suggesting that Treg activation is limited at disease sites.

### Attenuated IL-2 muteins enhance eTreg phenotype with reduced dependence on TCR signal

**Figure 7:**
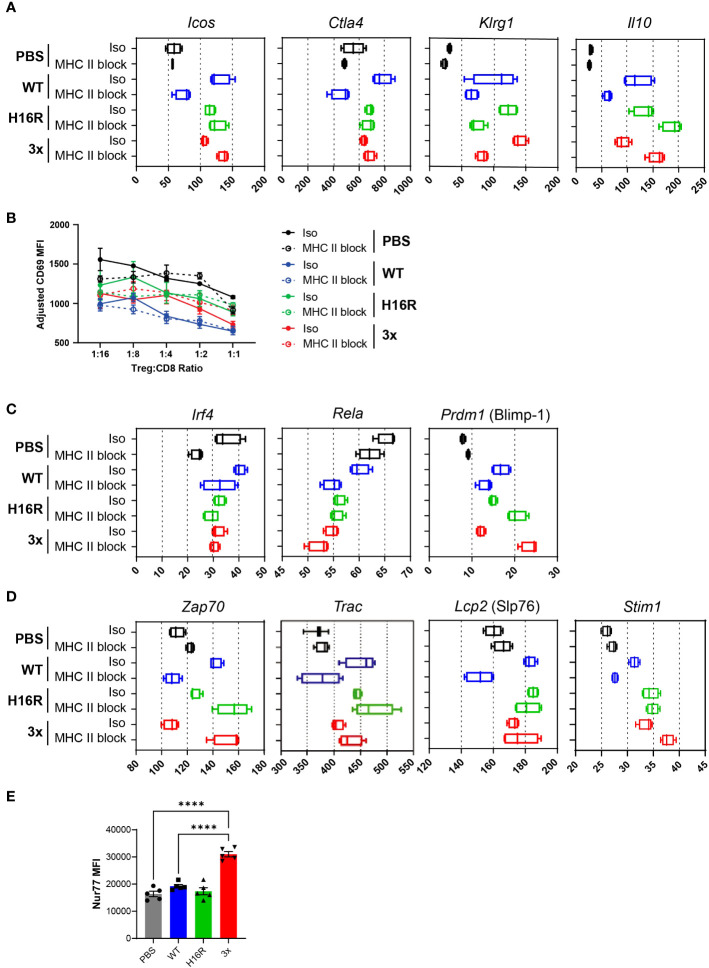
Enhancement of effector Treg phenotype and TCR response by IL-2. **(A)** Expression of eTreg signature genes in response to IL-2 with or without TCR blockade. The Fragments Per Kilobase of transcript per Million mapped reads (FPKM) values (x-axis) for representative eTreg signature genes in Tregs from mice dosed with PBS, WT IL-2, H16R, or 3x mutein with isotype control or MHC blocking antibody treatment are summarized in box plots. **(B)** Line plot summarizing the results from the *in vitro* suppression assay. Tregs purified from mice treated with IL-2 and antibody as indicated were co-cultured with purified autologous CD8 T cells at varying ratios as shown on the x-axis, in the presence of CD3/CD28 beads as a stimulating agent. CD8 T cell activation was quantified by CD69 expression and is shown as adjusted CD69 MFI on the y-axis. **(C)** Expression of known regulators of eTreg genes following IL-2 and TCR blockade treatments are shown individually as box plots. **(D)** Expression of genes encoding TCR signaling molecules following IL-2 and TCR blockade treatment are shown in box plots. **(E)** Nur77 expression in *ex vivo* Tregs. Tregs purified from splenocytes of mice treated with 25 μg of PBS, WT IL-2, H16R, or 3x mutein on day 3 were analyzed for Nur77 expression by flow cytometry. The mean fluorescence intensity (MFI) and SEM for each group (*n=5*) are summarized. Ordinary one-way ANOVA with Tukey’s multiple comparison’s test, ****p < 0.0001.

Functionally activated effector Tregs (eTregs) are distinguished from naïve central Tregs (cTregs) by multiple markers, some of which are directly involved in Treg’s ability to suppress inflammation and mediate tissue repair. As a readout of Treg functionality following IL-2 treatment, we assessed transcript levels for genes that are considered eTreg markers. As shown in **Figure 7A**, eTreg marker genes including *Icos*, *Ctla4*, *Klrg1, and Il10* were robustly upregulated in response to WT IL-2, and this response was largely abolished by the MHC II blocking treatment. Thus, WT IL-2 enhances eTreg phenotype in a TCR-dependent manner. The attenuated muteins H16R and 3x muteins also upregulated the eTreg marker genes. Interestingly, some genes induced by the attenuated muteins were not abrogated by the MHC II blockade (e.g., *Icos*, *Ctla4*, and *Il10*) and for some, such as *Il10 and Lag3* (not shown), the response was even elevated. These results suggested that the attenuated IL-2 muteins could enhance the eTreg phenotype even when the TCR signal was compromised.

To determine whether IL-2’s positive impact on eTreg gene signatures consequently led to enhanced Treg function, we performed an *in vitro* suppression assay. In a co-culture assay where purified CD8 T cell activation was assessed by CD69 expression, Tregs from mice treated with WT IL-2 showed a greater degree of suppression compared to Tregs from PBS-treated mice, suggesting that WT IL-2 enhanced Treg suppressive function *in vivo* (**Figure 7B**). Interestingly, MHC II blockade had little impact on the ability of IL-2 to enhance Treg function in this assay, suggesting that blocking the TCR signal *in vivo* did not impair Treg suppressive function in the short term up to 4 days. The two attenuated muteins also enhanced Treg function, although the level of suppression was intermediate between the WT IL2- and PBS-treated Tregs.

Molecular pathways implicated in the regulation of eTreg differentiation include the NF-kB pathway, triggered by engagement of one or more of the TNFRSFs expressed on Tregs (35), and the TCR pathway (8, 41), triggered when Tregs encounter the cognate antigen. Furthermore, several Foxp3 target genes including *Irf4*, *Rela*, and *Prdm1* (encoding Blimp-1) have been shown to confer specialized eTreg phenotype and function (42, 43). We interrogated whether IL-2 regulated the expression of *Irf4*, *Rela*, and *Prdm1* in Tregs *in vivo* and whether TCR signal was involved. The *Irf4* and *Rela* expression did not increase in response to WT IL-2 or the attenuated muteins, while *Prdm1* expression did, although the overall expression level was low (**Figure 7C**). These results suggest that IL-2 induces eTreg marker gene expression without directly increasing the expression levels of *Irf4*, *Rela*, and *Prdm1*.

We had observed that the turquoise module included genes that are known to drive TCR signaling. Thus, we considered the possibility that IL-2 enhanced TCR signaling in a positive feedback loop, as a mechanism of enhancing the eTreg gene signature downstream of TCR. In support of this hypothesis, *Zap70*, *Trac* (encoding TCR alpha), and *Slp76* expression increased in response to IL-2, and interestingly, the attenuated muteins were able to enhance their expression even when TCR signaling was blocked (**Figure 7D**). In fact, *Zap70* expression in response to the muteins was further amplified when TCR signaling was blocked. Additionally, several genes known to be involved in the calcium signaling pathway downstream of TCR, including Stim1 (**Figure 7D**), Orai1, and Nfatc3 (data not shown), were elevated, indicating that attenuated IL-2 signal may also enhance the calcium signaling pathway in Tregs.

Based on these results, we hypothesized that the IL-2 crosstalk with the TCR pathway directly enhanced Treg sensitivity to antigenic signal and adaptation of the eTreg phenotype. We tested this hypothesis by evaluating the expression of Nur77, which is a TCR-dependent gene known to be upregulated in circulating Tregs (9). Intracellular staining of Nur77 showed that Tregs from mice treated with 3x mutein indeed showed a strikingly elevated level of Nur77 (**Figure 7E**).

### Attenuated IL-2 muteins suppress tissue infiltration of pathogenic lymphocytes in a murine model of skin inflammation

Given that the IL-2 response gene signature was particularly enriched in the skin, we decided to evaluate the effects of IL-2 molecules in a mouse model of psoriasis. Human Fc-conjugated IL-2 molecules lose *in vivo* exposure as early as after the first dose (**Figure S6**), potentially due to anti-drug antibody (ADA) response; thus, we opted to evaluate the impact of IL-2 with a single dose in a short-term psoriasis model induced by application of the imiquimod (IMQ) cream.

**Figure 8:**
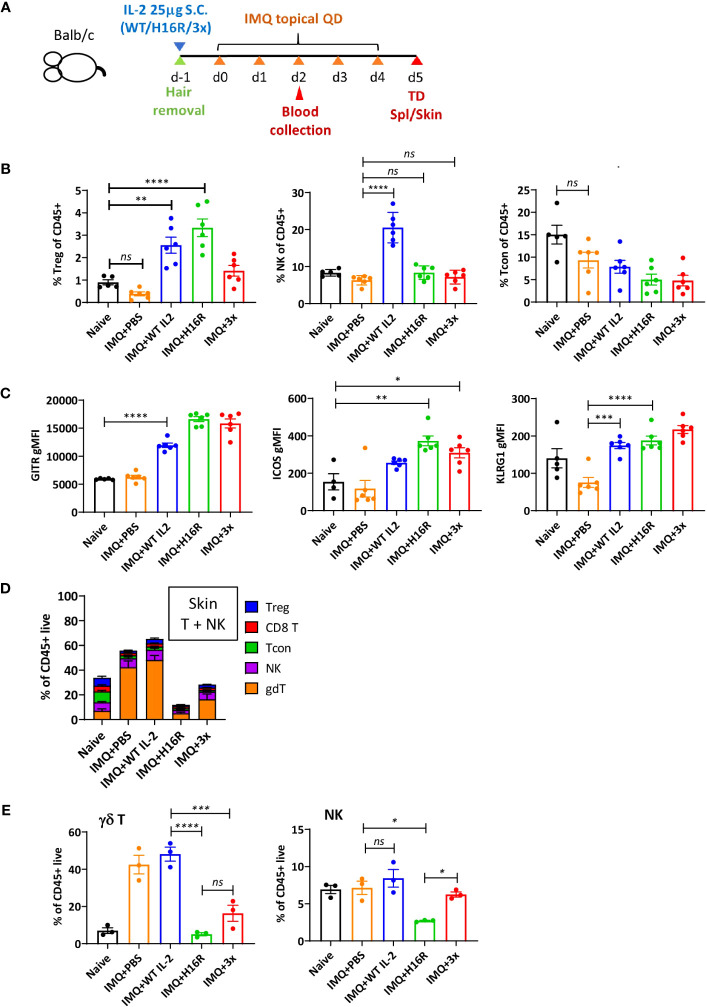
*In vivo* activity of attenuated IL-2 muteins in a mouse inflammatory skin disease model. **(A)** Study design. Female Balb/c mice were shaved on the back on day -1 and injected with IL-2 at 25 μg per mouse by s.c. Starting day 0, Aldara cream (3.125 mg per dose) was applied daily on the back and the ear for 5 doses and the study was terminated on d5. Blood was collected on day 3 post IL-2 dosing (d2 of disease induction) for flow analysis for T and NK cell response to IL-2. *n*=6 per group. **(B)** Treg, NK, and Tcon as percentage of CD45^+^ cells on day 3 blood. Each dot represents an animal, shown are the mean of the group and the error bars indicate SEM. One-way ANOVA analysis with Tukey’s multiple comparisons test, ****p < 0.0001, **p < 0.01, *ns*, not significant. Naive animals were shaved and handled the same way as disease animals, but they were not dosed with IL-2 or treated with Aldara cream. **(C)** eTreg marker expression in Tregs on d3 post IL-2 dosing in blood. GITR, ICOS, and KLRG1 expression was determined by flow cytometric analysis and the expression level was summarized as gMFI in total Treg population for each treatment group. One-way ANOVA with Tukey’s multiple comparisons test, ****p < 0.0001, ***p < 0.001, **p < 0.01, *p < 0.05. **(D)** T and NK cell composition in the skin on day 5 at take down. The breakdown of total T and NK cells, shown as the percentages of Treg, CD8 T, Tcon (Foxp3^-^ CD4 T), NK, and γδ T cells of CD45^+^ live cells in the skin on day 5, are summarized for the different treatment and disease groups. **(E)** The percentages of γδ T and NK cells among CD45^+^ live cells in the skin on day 5. The data in **(D)** are presented individually for γδ T and NK cells. One-way ANOVA with Tukey’s multiple comparisons test, ****p < 0.0001, ***p < 0.001, **p < 0.01, *p < 0.05. For **(D, E)**, *n*=3 per group. Each symbol represents an animal.

We administered a prophylactic dose of IL-2 on day -1 and induced disease by daily application of the IMQ cream for 5 days (**Figure 8A**). On day 2 (3 days after IL-2 treatment), we detected a robust increase in the percentage of Tregs in blood following treatment with WT IL-2 or H16R, while 3x mutein showed a trend of increase (**Figure 8B**). The attenuated muteins induced negligible NK and Tcon responses, but WT IL-2 induced significant expansion of NK cells. These results confirmed that attenuated IL-2 muteins induced Treg expansion *in vivo* with increased Treg selectivity even in an inflammatory setting. Furthermore, Tregs in mice treated with IL-2 expressed higher levels of activation and eTreg markers, including *Gitr*, *Icos*, and *Klrg1*, validating that IL-2 induces eTreg marker gene expression at the protein level (**Figure 8C**).

With a single dose administration of IL-2, we did not observe disease improvement measured by body weight and skin thickness (**Figures S7A, B**). In this model, the skin pathology is characterized by immune cell infiltration, with gamma delta (γδ) T cells specifically implicated as the main driver of disease (44). On day 5 at the end of the study, the percentage of γδ T cells was strikingly elevated, while the percentages of Tregs and Tcons were reduced, in the skin of diseased (i.e., IMQ-treated) compared to naïve mice (**Figure 8D**). Mice treated with WT IL-2 maintained these same disease-associated changes, while H16R and 3x mutein treatment suppressed the accumulation of γδ T and NK cells in the skin (**Figure 8E**). Evaluation of Ki67 showed that the skin γδ T and NK cells proliferated similarly in all diseased animals regardless of the IL-2 treatment (**Figure S7C**). These results suggest that IL-2 promotes expansion of circulating Tregs, enhances the eTreg phenotype and suppresses immune cell (including γδ T and NK cells) trafficking to the inflamed tissue. In our study, the two attenuated muteins demonstrated improved Treg selectivity and ability to suppress pathogenic cell infiltration compared to WT IL-2 in a disease setting.

## Discussion

IL-2 is required for Treg proliferation, survival, and function [reviewed by (45)]. Attenuated IL-2 muteins with weaker affinity to IL2Rβ and/or IL2Rγ demonstrate a greater selectivity for Tregs largely because of the increased dependence on CD25 for binding and because Tregs are more sensitive to lower IL-2 signal than other lymphocytes [review by (46)]. An important consideration for clinical utilization of attenuated IL-2 platforms is the minimal IL-2 signaling threshold required to support the Treg responses capable of establishing and maintaining immune homeostasis. In this study, we generated further insight into this question by comparing the Treg responses to WT IL-2 and two attenuated IL-2 muteins in mice.

*In vitro*, WT IL-2 and the muteins ranked consistently (WT>H16R>3x>D20W) in terms of activity to induce STAT5 activation, proliferation, activation marker expression, and suppressive function in human Tregs. We confirmed that the rank order of activity of these human IL-2 molecules was conserved in mouse Tregs as measured by pSTAT5 readout *in vitro*.

A single-dose administration of these IL-2 molecules in mice generated a dose-dependent expansion of mouse Tregs. Unexpectedly, besides the enhanced Treg selectivity, the attenuated muteins generated a disproportionately robust Treg expansion, even exceeding the level of expansion achieved by WT IL-2. Transcriptomic and pathway analyses of differentially expressed genes in Tregs treated with WT IL-2, H16R, or 3x mutein showed overlapping gene signatures, pointing to a general ‘IL-2 signature response’ in Tregs. These genes included activation pathways that are relevant for programming Tregs’ fitness and subsequent responses. IL-2 broadly induced genes involved in immune responses (including cytokine receptor genes such as *Il10rb*, *Ifnar*, *Il1rl1*, and *Il9r*), cell migration responses (captured by increased expression of chemokine receptors including *Ccr8* and tissue-targeting receptors such as *Itgae*, *Itgb1*, and *Itgb7*), cell cycle regulation, and TNFRSF members (e.g., *Cd27*, *Cd30*, *Tnfrsf4*, and *Tnfrsf1b*). This was consistent with the known role of IL-2 on Tregs, but we noted differences in the response patterns in both the induced and repressed genes between WT IL-2 and the attenuated muteins, suggesting that the attenuated IL-2 muteins induced qualitatively distinct transcriptional response compared to that induced by WT IL-2. In interpreting our data, we acknowledge the possibility that some of these differences reflect indirect contribution of non-Tregs responding to IL-2 *in vivo*; nonetheless we took a simplistic approach with an assumption that these interactions comprise normal immune regulatory mechanisms that define the *in vivo* response of Tregs.

The unexpectedly robust effect of the far-attenuated 3x mutein led us to hypothesize that the weak IL-2 signal synergized with additional signals present *in vivo*. Since it has been established that Tregs require both IL-2 and TCR signal for maintenance and function *in vivo*, we investigated the impact of reduced TCR signaling by blocking MHC II with an antibody treatment. Treg expansion, both in terms of proportion and absolute number, was almost completely abrogated, while Treg activation, as read out by induction of multiple markers (e.g., Ki67, Gitr), was unaffected. Furthermore, attenuated muteins elicited a disproportionately robust response that was comparable to or even better than that elicited by WT IL-2, even when TCR signal was reduced.

Further analysis by WGCNA in this system revealed modules of genes that were coregulated in IL2- and/or TCR-dependent manners. We focused on pathway analysis of these modules and highlighted two modules based on the significance of association with predicted pathways. The turquoise module represented the largest module with strong association with multiple pathways that were positively regulated in an IL2-dependent manner. Interestingly, the turquoise module response was sensitive to TCR blockade for WT IL-2 but not for attenuated muteins. This response suggested that attenuated IL-2 muteins amplified the turquoise module pathways with reduced dependence on the TCR signal. The green module was associated with TCR-dependent induction regardless of IL-2 and highlighted the MAPK and TNFRSF/NF-kB pathways, which suggests that the antigenic signal is the main activator of these pathways. The disparate contribution of TCR signal in this context supports a model wherein TCR signal by itself contributes to limited aspects of Treg activation, but cooperation with IL-2 dramatically broadens its impact. Our findings further suggest that IL-2 can potentiate and amplify the TCR signaling in Tregs, at least in part by increasing the expression of genes that constitute the TCR complex and the calcium signaling pathway. The WGCNA analysis did not reveal module(s) of genes that were strictly IL2-dependent and TCR-independent, which we had initially hypothesized induced Treg activation response distinctly from Treg expansion response. Nonetheless, the turquoise module showed this pattern in response to the attenuated muteins, indicating that attenuated IL-2 muteins act on Tregs with modified requirements for synergy with other signaling pathways. Additionally, it remains likely that IL-2 synergizes with other stimuli *in vivo*.

The turquoise module genes showed alignment with Treg signatures associated with NLT, suppressive function, and eTreg states. Here we propose that the IL2-mediated activation of the turquoise module pathways drives the eTreg response. Treg expansion, in contrast, was sensitive to MHC II blocking treatment for all IL-2 molecules, so we hypothesize that the green module pathways are required for durable Treg expansion and accumulation *in vivo*. Whether this is through increased proliferation and/or reduced cell death requires further study.

Splenic Tregs in IL2-treated mice displayed amplified gene signatures that overlapped with tissue-resident Tregs (skin and colon Tregs). This is consistent with a model wherein IL-2 increases the migratory behavior of Tregs as they circulate actively between LTs and NLTs. In our study, Tregs responded largely to the synergistic effects of IL-2 and TCR signals (as captured by the turquoise module genes); thus tissue-associated antigens likely play a major role in imparting tissue-specific gene signatures involved in Treg activation and migration in response to IL-2.

In a short-term inflammatory skin disease model, a single dose of attenuated IL-2 muteins promoted expansion of Tregs with enhanced selectivity, Treg adaptation of the eTreg phenotype, and suppression of pathogenic cell (γδ T and NK cells) infiltration into the skin. Interestingly, suppression of tissue infiltration of γδ T and NK cells was not accompanied by increased accumulation of Tregs in the tissue despite increased representation in the blood. This suggests that Tregs may exert their suppressive effects remotely. We showed that WT IL-2 also expanded Tregs, but the effect was not as selective, and it did not suppress tissue infiltration of γδ T and NK cells. These data showed that the attenuated muteins generated functional Treg response that was superior to that induced by WT IL-2 at an equivalently high dose.

The attenuated IL-2 muteins demonstrated robust activity *in vivo* that were incongruent with their *in vitro* activity and induced modified transcriptional and pathway responses compared to WT IL-2. Their amplified *in vivo* activity may have resulted at least in part from the reduced receptor-mediated clearance and consequently increased duration in circulation (29, 47). The measured *in vivo* PK parameters indeed showed that the attenuated IL-2 muteins, particularly the far-attenuated 3x mutein, exhibited decreased clearance and increased half-life compared to WT IL-2 or H16R, and also generated a sustained pSTAT5 response. Previously it was shown that sustained IL2R signaling was required to expand Tregs *in vivo* when comparing short-acting recombinant IL-2 with a long-acting IL-2/CD25 complex, and Treg transcriptomic data highlighted pathways associated with TCR and IL-2R signaling (48). Our data are consistent with these results, but we further showed that the attenuated IL-2 muteins generated gene responses distinct from WT IL-2, including upregulation of genes involved in the TCR signaling machinery such as Slp76 and genes involved in the calcium signaling pathway such as Stim1 and Orai1, even when the TCR signal is blocked. We propose that these changes led to enhanced TCR signaling as evidenced by increased Nur77 in circulating Tregs, which in turn further amplified Treg activation and expansion. An interesting therapeutic implication is that Treg activation may be achievable in a relatively tissue antigen-agnostic manner with attenuated IL-2 muteins, which could be used in a broad spectrum of auto-immune diseases.

Alternatively, strong vs. weak IL-2 signal may enforce Tregs to adapt distinct cell phenotypes, as had been described for CD8 T cells that were shown to adapt distinct responses depending on the strength and duration of the IL-2 signal (49, 50). An earlier model posited that a “stronger” IL-2 signal led to terminally differentiated effector CTL fate while “weaker” IL-2 signal led to long-term memory cells, and this cell fate choice was accompanied by different transcriptional responses. More recently, it was shown that the TCR signal strength determined the requirement for IL-2 signal in forming a CD8 T cell memory response (51). Our data suggest that Treg responses are similarly shaped by the combined effects of TCR and IL-2 signals, and that in Tregs the IL-2 signal strength influences the requirement for the TCR signal.

In this study, we showed that Tregs rely heavily on the synergistic effects of IL-2 and TCR signals for homeostatic maintenance and activation. These pathways interact closely rather than act in parallel, and their integrated output is likely to set the threshold of activation and duration of response. In the half life-extended format, attenuated IL-2 muteins generated reduced peak STAT5 signal compared to WT IL-2 but still induced robust Treg expansion and suppressed pathogenic cell infiltration better than WT IL-2. These effects were accompanied by signs of enhanced TCR signal in circulating Tregs, which likely impacted their phenotype and function. Further studies are required to understand how additional pathways may be integrated to regulate Treg responses *in vivo* and to apply this knowledge for improved rational design of Treg-targeted and IL2-based therapies.

## Data availability statement

All raw and processed RNA-seq data from this study have been deposited in the NCBI Gene Expression Omnibus (GEO; https://www.ncbi.nim.nih.gov/geo/) with accession no. GSE216130 (https://www.ncbi.nlm.nih.gov/geo/query/acc.cgi?acc=GSE216130).

## Ethics statement

Ethical approval was not required for the study involving humans in accordance with the local legislation and institutional requirements. Written informed consent to participate in this study was not required from the participants or the participants’ legal guardians/next of kin in accordance with the national legislation and the institutional requirements. The animal study was approved by Institutional Animal Care and Use Committee (IACUC) of Amgen. The study was conducted in accordance with the local legislation and institutional requirements.

## Author contributions

SM: Data curation, Formal Analysis, Investigation, Methodology, Validation, Visualization, Writing – original draft, Writing – review & editing, Software. MiS: Formal Analysis, Writing – original draft, Writing – review & editing, Data curation, Methodology, Visualization. AG: Data curation, Formal Analysis, Methodology, Writing – review & editing, Investigation, Validation, Visualization. RS: Data curation, Formal Analysis, Methodology, Validation, Writing – review & editing, Software, Visualization. AS: Data curation, Formal Analysis, Methodology, Validation, Writing – review & editing, Visualization. RH: Data curation, Formal Analysis, Methodology, Validation, Visualization, Writing – review & editing, Supervision, Writing – original draft. WL: Data curation, Formal Analysis, Methodology, Supervision, Validation, Visualization, Writing – original draft, Writing – review & editing, Investigation, Project administration. HW: Data curation, Formal Analysis, Methodology, Project administration, Supervision, Validation, Visualization, Writing – original draft, Writing – review & editing. SY: Data curation, Formal Analysis, Methodology, Writing – review & editing. SL: Data curation, Methodology, Writing – review & editing. ES: Data curation, Methodology, Writing – review & editing. TC: Data curation, Methodology, Writing – review & editing, Formal Analysis, Visualization. MaS: Data curation, Methodology, Writing – review & editing. HZ: Data curation, Methodology, Writing – review & editing, Formal Analysis, Validation. C-ML: Data curation, Methodology, Writing – review & editing, Funding acquisition, Resources, Supervision, Writing – original draft. AC: Data curation, Methodology, Resources, Supervision, Writing – review & editing, Formal Analysis, Investigation, Project administration. XL: Data curation, Formal Analysis, Investigation, Methodology, Project administration, Resources, Supervision, Writing – review & editing, Software, Validation, Visualization, Writing – original draft. SS: Data curation, Formal Analysis, Investigation, Methodology, Project administration, Resources, Supervision, Validation, Visualization, Writing – original draft, Writing – review & editing, Conceptualization, Funding acquisition.

## References

[1] Shevach EM. Mechanisms of foxp3+ T regulatory cell-mediated suppression. Immunity (2009) 30(5):636–45. doi: 10.1016/j.immuni.2009.04.010 19464986

[2] Campbell C Rudensky A. Roles of regulatory T cells in tissue pathophysiology and metabolism. Cell Metab (2020) 31(1):18–25. doi: 10.1016/j.cmet.2019.09.010 31607562 PMC7657366

[3] Bennett CL Christie J Ramsdell F Brunkow ME Ferguson PJ Whitesell L. The immune dysregulation, polyendocrinopathy, enteropathy, X-linked syndrome (IPEX) is caused by mutations of FOXP3. Nat Genet (2001) 27(1):20–1. doi: 10.1038/83713 11137993

[4] Zheng Y Rudensky AY. Foxp3 in control of the regulatory T cell lineage. Nat Immunol (2007) 8(5):457–62. doi: 10.1038/ni1455 17440451

[5] Brunkow ME Jeffery EW Hjerrild KA Paeper B Clark LB Yasayko SA. Disruption of a new forkhead/winged-helix protein, scurfin, results in the fatal lymphoproliferative disorder of the scurfy mouse. Nat Genet (2001) 27(1):68–73. doi: 10.1038/83784 11138001

[6] Josefowicz SZ Lu LF Rudensky AY. Regulatory T cells: mechanisms of differentiation and function. Annu Rev Immunol (2012) 30:531–64. doi: 10.1146/annurev.immunol.25.022106.141623 PMC6066374 22224781

[7] Chinen T Kannan AK Levine AG Fan X Klein U Zheng Y. An essential role for the IL-2 receptor in Treg cell function. Nat Immunol (2016) 17(11):1322–33. doi: 10.1038/ni.3540 PMC5071159 27595233

[8] Levine AG Arvey A Jin W Rudensky AY. Continuous requirement for the TCR in regulatory T cell function. Nat Immunol (2014) 15(11):1070–8. doi: 10.1038/ni.3004 PMC4205268 25263123

[9] Moran AE Holzapfel KL Xing Y Cunningham NR Maltzman JS Punt J. T cell receptor signal strength in Treg and iNKT cell development demonstrated by a novel fluorescent reporter mouse. J Exp Med (2011) 208(6):1279–89. doi: 10.1084/jem.20110308 PMC3173240 21606508

[10] Smigiel KS Richards E Srivastava S Thomas KR Dudda JC Klonowski KD. CCR7 provides localized access to IL-2 and defines homeostatically distinct regulatory T cell subsets. J Exp Med (2014) 211(1):121–36. doi: 10.1084/jem.20131142 PMC3892972 24378538

[11] Liu Z Gerner MY Van Panhuys N Levine AG Rudensky AY Germain RN. Immune homeostasis enforced by co-localized effector and regulatory T cells. Nature (2015) 528(7581):225–30. doi: 10.1038/nature16169 PMC4702500 26605524

[12] Grasshoff H Comduhr S Monne LR Muller A Lamprecht P Riemekasten G. Low-dose IL-2 therapy in autoimmune and rheumatic diseases. Front Immunol (2021) 12:648408. doi: 10.3389/fimmu.2021.648408 33868284 PMC8047324

[13] Dixit N Fanton C Langowski JL Kirksey Y Kirk P Chang T. NKTR-358: A novel regulatory T-cell stimulator that selectively stimulates expansion and suppressive function of regulatory T cells for the treatment of autoimmune and inflammatory diseases. J Transl Autoimmun (2021) 4:100103. doi: 10.1016/j.jtauto.2021.100103 34041473 PMC8141531

[14] Tchao N Gorski K Yuraszeck T Sohn S Ishida K Wong H. PS7:135 Amg 592 is an investigational il-2 mutein that induces highly selective expansion of regulatory t cells. Lupus Sci Med (2018) 5(Suppl 1):A102–A. doi: 10.1136/lupus-2018-abstract.178

[15] Cully M. T cell-regulating therapies for autoimmune diseases take FDA rejection in stride. Nat Rev Drug Discovery (2021) 20:655–7. doi: 10.1038/d41573-021-00137-0 34363024

[16] Ghelani A Bates D Conner K Wu MZ Lu J Hu YL. Defining the threshold IL-2 signal required for induction of selective treg cell responses using engineered IL-2 muteins. Front Immunol (2020) 11:1106. doi: 10.3389/fimmu.2020.01106 32582190 PMC7291599

[17] Zhang Y Huo M Zhou J Xie S. PKSolver: An add-in program for pharmacokinetic and pharmacodynamic data analysis in Microsoft Excel. Comput Methods Programs Biomed (2010) 99(3):306–14. doi: 10.1016/j.cmpb.2010.01.007 20176408

[18] Li B Dewey CN. RSEM: accurate transcript quantification from RNA-Seq data with or without a reference genome. BMC Bioinf (2011) 12:323. doi: 10.1186/1471-2105-12-323 PMC3163565 21816040

[19] Hu J Ge H Newman M Liu K. OSA: a fast and accurate alignment tool for RNA-Seq. Bioinformatics (2012) 28(14):1933–4. doi: 10.1093/bioinformatics/bts294 22592379

[20] Love MI Huber W Anders S. Moderated estimation of fold change and dispersion for RNA-seq data with DESeq2. Genome Biol (2014) 15(12):550. doi: 10.1186/s13059-014-0550-8 25516281 PMC4302049

[21] Gu Z Eils R Schlesner M. Complex heatmaps reveal patterns and correlations in multidimensional genomic data. Bioinformatics (2016) 32(18):2847–9. doi: 10.1093/bioinformatics/btw313 27207943

[22] Langfelder P Horvath S. WGCNA: an R package for weighted correlation network analysis. BMC Bioinf (2008) 9:559. doi: 10.1186/1471-2105-9-559 PMC2631488 19114008

[23] Langfelder P Horvath S. Fast R functions for robust correlations and hierarchical clustering. J Stat Software (2012) 46(11). doi: 10.18637/jss.v046.i11 PMC3465711 23050260

[24] Yu G Wang LG Han Y He QY. clusterProfiler: an R package for comparing biological themes among gene clusters. OMICS (2012) 16(5):284–7. doi: 10.1089/omi.2011.0118 PMC3339379 22455463

[25] Yu G He QY. ReactomePA: an R/Bioconductor package for reactome pathway analysis and visualization. Mol Biosyst (2016) 12(2):477–9. doi: 10.1039/C5MB00663E 26661513

[26] Lu DR Wu H Driver I Ingersoll S Sohn S Wang S. Dynamic changes in the regulatory T-cell heterogeneity and function by murine IL-2 mutein. Life Sci Alliance (2020) 3(5). doi: 10.26508/lsa.201900520 PMC7156283 32269069

[27] Miragaia RJ Gomes T Chomka A Jardine L Riedel A Hegazy AN. Single-cell transcriptomics of regulatory T cells reveals trajectories of tissue adaptation. Immunity (2019) 50(2):493–504 e7. doi: 10.1016/j.immuni.2019.01.001 30737144 PMC6382439

[28] Hao Y Hao S Andersen-Nissen E Mauck WM 3rd Zheng S Butler A. Integrated analysis of multimodal single-cell data. Cell (2021) 184(13):3573–87 e29. doi: 10.1016/j.cell.2021.04.048 34062119 PMC8238499

[29] de Picciotto S DeVita N Hsiao CJ Honan C Tse SW Nguyen M. Selective activation and expansion of regulatory T cells using lipid encapsulated mRNA encoding a long-acting IL-2 mutein. Nat Commun (2022) 13(1):3866. doi: 10.1038/s41467-022-31130-9 35790728 PMC9256694

[30] Ottolenghi A Bolel P Sarkar R Greenshpan Y Iraqi M Ghosh S. Life-extended glycosylated IL-2 promotes Treg induction and suppression of autoimmunity. Sci Rep (2021) 11(1):7676. doi: 10.1038/s41598-021-87102-4 33828163 PMC8027413

[31] Zhang R Huynh A Whitcher G Chang J Maltzman JS Turka LA. An obligate cell-intrinsic function for CD28 in Tregs. J Clin Invest (2013) 123(2):580–93. doi: 10.1172/JCI65013 PMC3561819 23281398

[32] Kornete M Sgouroudis E Piccirillo CA. ICOS-dependent homeostasis and function of Foxp3+ regulatory T cells in islets of nonobese diabetic mice. J Immunol (2012) 188(3):1064–74. doi: 10.4049/jimmunol.1101303 22227569

[33] Elpek KG Yolcu ES Franke DD Lacelle C Schabowsky RH Shirwan H. *Ex vivo* expansion of CD4+CD25+FoxP3+ T regulatory cells based on synergy between IL-2 and 4-1BB signaling. J Immunol (2007) 179(11):7295–304. doi: 10.4049/jimmunol.179.11.7295 18025172

[34] Kumar P Alharshawi K Bhattacharya P Marinelarena A Haddad C Sun Z. Soluble OX40L and JAG1 Induce Selective Proliferation of Functional Regulatory T-Cells Independent of canonical TCR signaling. Sci Rep (2017) 7:39751. doi: 10.1038/srep39751 28045060 PMC5206631

[35] Vasanthakumar A Liao Y Teh P Pascutti MF Oja AE Garnham AL. The TNF receptor superfamily-NF-kappaB axis is critical to maintain effector regulatory T cells in lymphoid and non-lymphoid tissues. Cell Rep (2017) 20(12):2906–20. doi: 10.1016/j.celrep.2017.08.068 28889989

[36] Stremska ME Jose S Sabapathy V Huang L Bajwa A Kinsey GR. IL233, A novel IL-2 and IL-33 hybrid cytokine, ameliorates renal injury. J Am Soc Nephrol (2017) 28(9):2681–93. doi: 10.1681/ASN.2016121272 PMC5576940 28539382

[37] Zheng SG Wang J Wang P Gray JD Horwitz DA. IL-2 is essential for TGF-beta to convert naive CD4+CD25- cells to CD25+Foxp3+ regulatory T cells and for expansion of these cells. J Immunol (2007) 178(4):2018–27. doi: 10.4049/jimmunol.178.4.2018 17277105

[38] De Rosa V Procaccini C Cali G Pirozzi G Fontana S Zappacosta S. A key role of leptin in the control of regulatory T cell proliferation. Immunity (2007) 26(2):241–55. doi: 10.1016/j.immuni.2007.01.011 17307705

[39] Krämer A Green J Pollard J Tugendreich S. Causal analysis approaches in Ingenuity Pathway Analysis. Bioinformatics (2014) 30(4):523–30. doi: 10.1093/bioinformatics/btt703 PMC3928520 24336805

[40] Reynolds G Vegh P Fletcher J Poyner EFM Stephenson E Goh I. Developmental cell programs are co-opted in inflammatory skin disease. Science (2021) 371(6527). doi: 10.1126/science.aba6500 PMC7611557 33479125

[41] Vahl JC Drees C Heger K Heink S Fischer JC Nedjic J. Continuous T cell receptor signals maintain a functional regulatory T cell pool. Immunity (2014) 41(5):722–36. doi: 10.1016/j.immuni.2014.10.012 25464853

[42] Vasanthakumar A Moro K Xin A Liao Y Gloury R Kawamoto S. The transcriptional regulators IRF4, BATF and IL-33 orchestrate development and maintenance of adipose tissue-resident regulatory T cells. Nat Immunol (2015) 16(3):276–85. doi: 10.1038/ni.3085 25599561

[43] Zheng Y Chaudhry A Kas A deRoos P Kim JM Chu TT. Regulatory T-cell suppressor program co-opts transcription factor IRF4 to control T(H)2 responses. Nature (2009) 458(7236):351–6. doi: 10.1038/nature07674 PMC2864791 19182775

[44] Hartwig T Zwicky P Schreiner B Yawalkar N Cheng P Navarini A. Regulatory T cells restrain pathogenic T helper cells during skin inflammation. Cell Rep (2018) 25(13):3564–72 e4. doi: 10.1016/j.celrep.2018.12.012 30590032

[45] Abbas AK Trotta E D Marson A Bluestone JA. Revisiting IL-2: Biology and therapeutic prospects. Sci Immunol (2018) 3(25). doi: 10.1126/sciimmunol.aat1482 29980618

[46] Mitra S Leonard WJ. Biology of IL-2 and its therapeutic modulation: Mechanisms and strategies. J Leukoc Biol (2018) 103(4):643–55. doi: 10.1002/JLB.2RI0717-278R 29522246

[47] Khoryati L Pham MN Sherve M Kumari S Cook K Pearson J. An IL-2 mutein engineered to promote expansion of regulatory T cells arrests ongoing autoimmunity in mice. Sci Immunol (2020) 5(50). doi: 10.1126/sciimmunol.aba5264 PMC7643170 32817295

[48] Moro A Gao Z Wang L Yu A Hsiung S Ban Y. Dynamic transcriptional activity and chromatin remodeling of regulatory T cells after varied duration of interleukin-2 receptor signaling. Nat Immunol (2022) 23(5):802–13. doi: 10.1038/s41590-022-01179-1 PMC9106907 35449416

[49] Pipkin ME Sacks JA Cruz-Guilloty F Lichtenheld MG Bevan MJ Rao A. Interleukin-2 and inflammation induce distinct transcriptional programs that promote the differentiation of effector cytolytic T cells. Immunity (2010) 32(1):79–90. doi: 10.1016/j.immuni.2009.11.012 20096607 PMC2906224

[50] Kalia V Sarkar S Subramaniam S Haining WN Smith KA Ahmed R. Prolonged interleukin-2Ralpha expression on virus-specific CD8+ T cells favors terminal-effector differentiation *in vivo* . Immunity (2010) 32(1):91–103. doi: 10.1016/j.immuni.2009.11.010 20096608

[51] Chin SS Guillen E Chorro L Achar S Ng K Oberle S. T cell receptor and IL-2 signaling strength control memory CD8(+) T cell functional fitness via chromatin remodeling. Nat Commun (2022) 13(1):2240. doi: 10.1038/s41467-022-29718-2 35474218 PMC9042912

