# Supplementary material for: Attenuated IL-2 muteins leverage the TCR signal to enhance regulatory T cell homeostasis and response in vivo
Source: Front Immunol. 2023 Sep 22;14:1257652. doi: 10.3389/fimmu.2023.1257652 (PMC10556740; doi:10.3389/fimmu.2023.1257652)
Supplement: Supplementary file 1 [file DataSheet_1.pdf]

A.

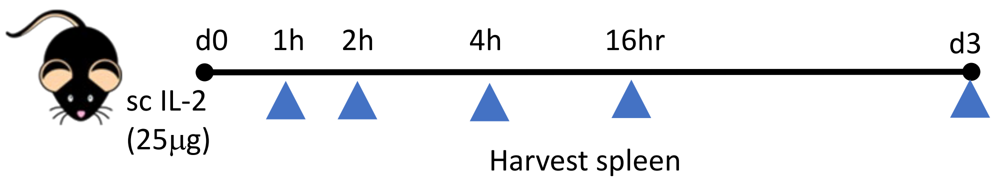

B.

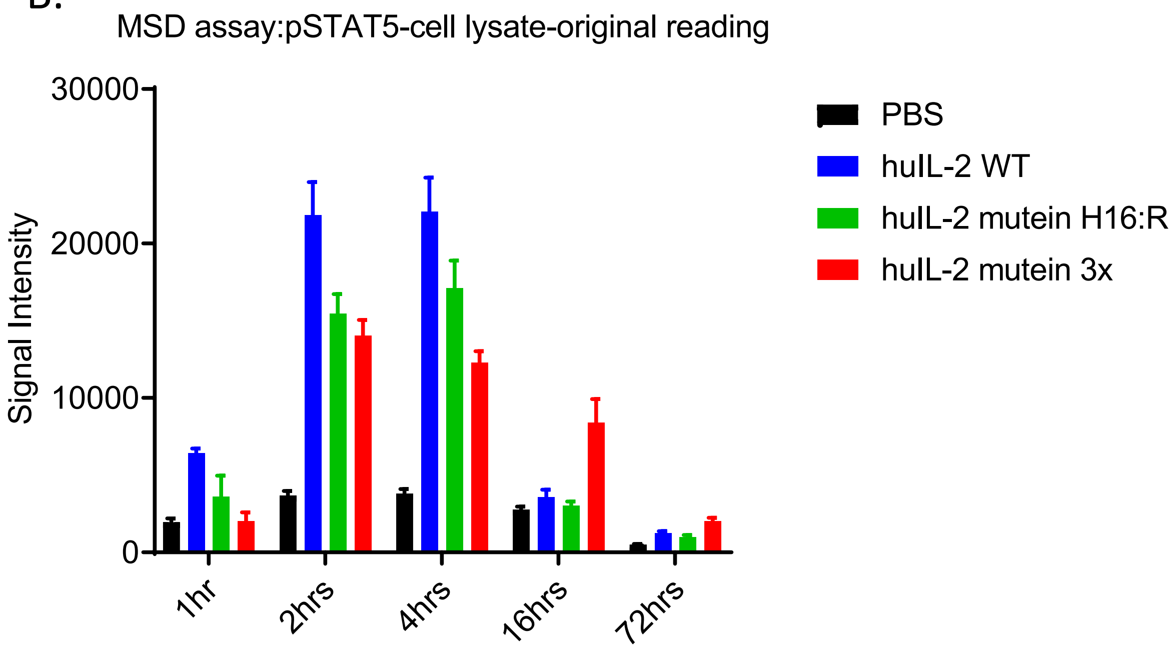

Fig. S1. The kinetics of the phospho-STAT5 signal following a single dose administration of IL-2 in mice. A, the study design. B, The raw values of the pSTAT5 MSD signal, measured at indicated times after dosing with PBS, WT IL-2, H16R, or 3x mutein, are shown as the mean of the group (*n=5*). The error bars represent the SEM.

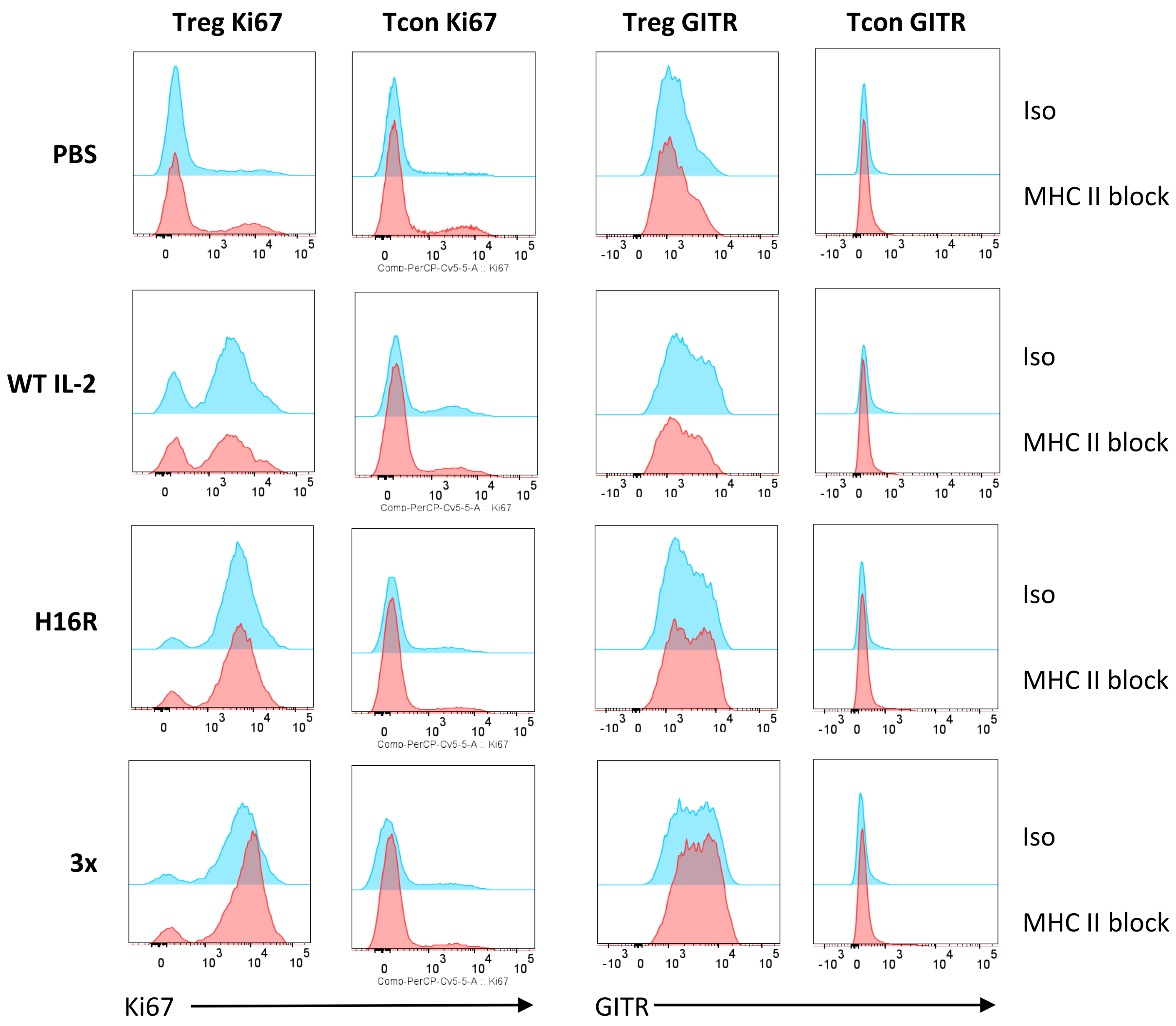

Fig. S2. Treg and Tcon response to WT IL-2 and the attenuated muteins in combination with the MHC II blockade treatment in vivo.   Representative histograms showing the Ki67 and GITR expression in splenic Treg (Foxp3+ CD25+ CD4 T) and Tcon (Foxp3- CD4 T) cells from mice treated with PBS, WT IL-2, H16R, or 3x mutein and isotype control (blue) or MHC II blocking (red) antibody are shown.

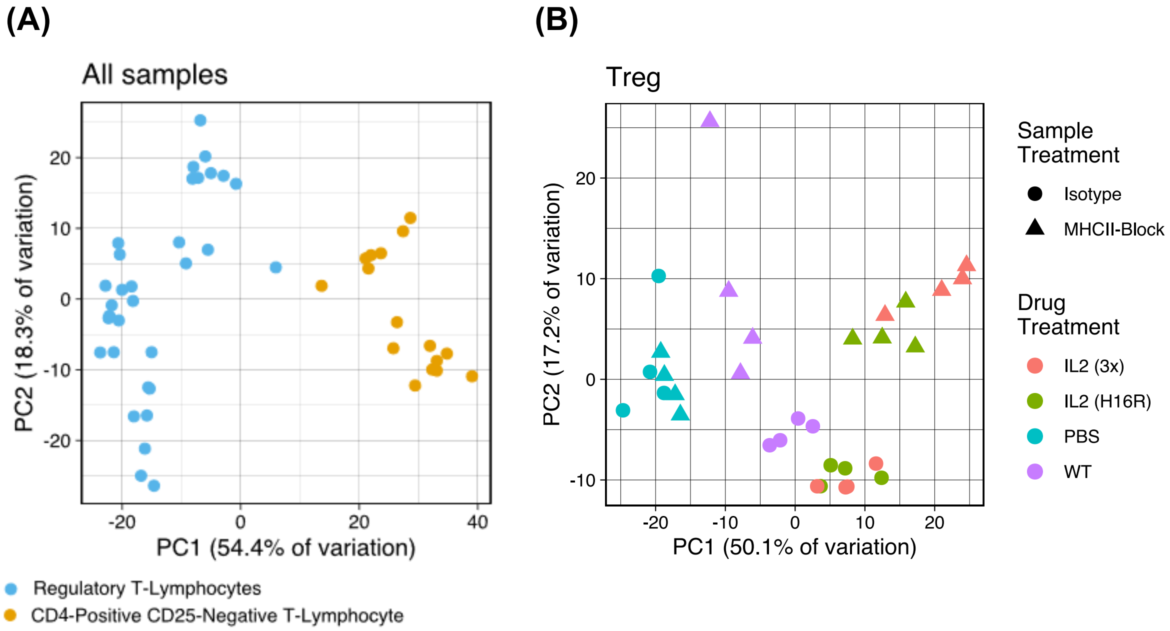

Fig. S3. The two-dimensional principal subspace of samples based on bulk RNA-Seq mRNA expression. (A) PCA of the 32 Treg (blue circle) and 16 CD25-Tcon(brown circle) samples. (B) PCA of the Treg samples only. Isotype antibody treated samples are represented by the circles and MHC II blocking antibody-treated samples by the triangles. The various IL-2 treatments are indicated by different colors, PBS (turquoise), WT IL-2 (purple), H16R (green), and 3x (red). Each treatment group contained 4 samples.

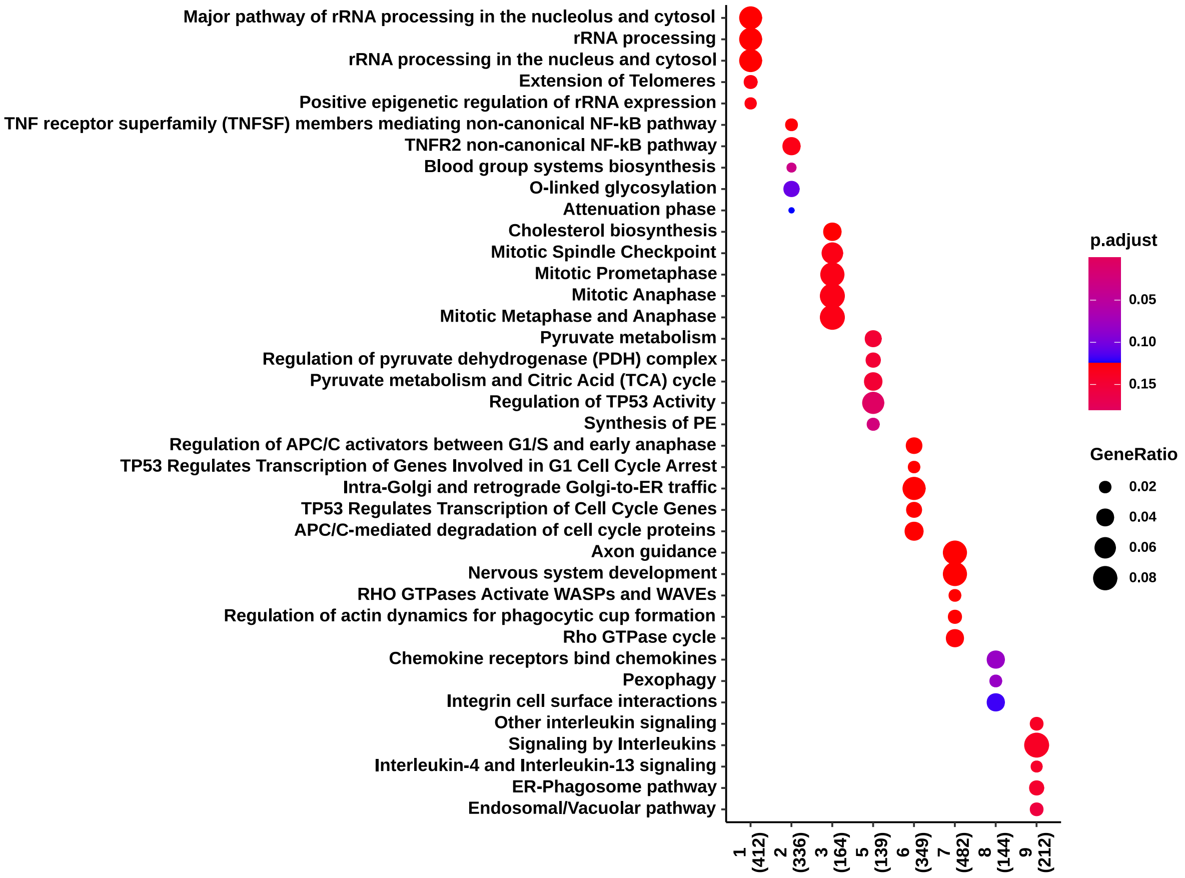

Fig. S4. Comparison of top enriched Reactome pathways associated with individual clusters shown in figure 3B. The color of dots represents increasing significance of the enriched pathways from blue to red and the size of the dot reflects the GeneRatio (Proportion of DEGs in a specific cluster that associated with the given Reatome Term). Number of DEGs analyzed in the gene sets are in parentheses.

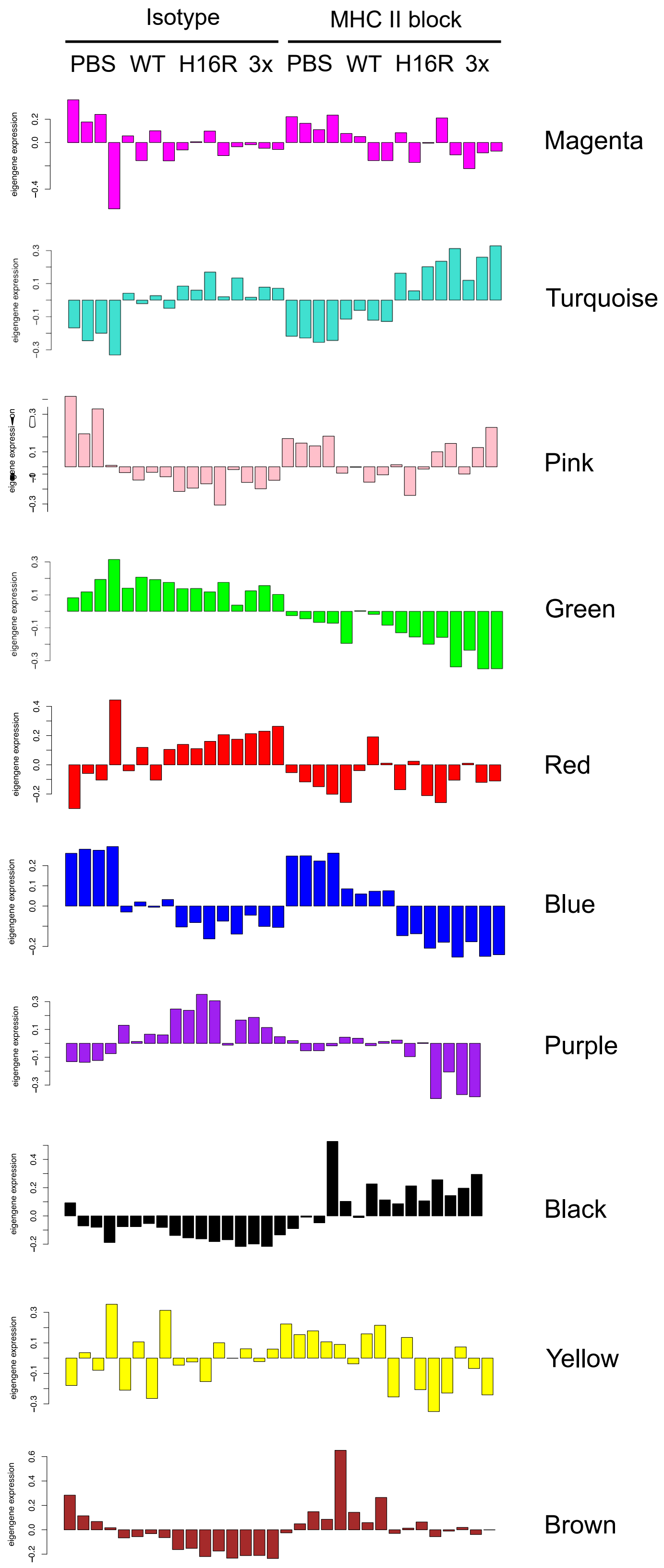

Fig. S5. Eigengene bar graphs for the WGCNA modules identified in Fig. 4. The module eigengene values of individual samples are defined as the first principal component of a given module to represent the gene expression profiles in a module. Each bar represents a sample, with *n=4* per treatment group indicated above.

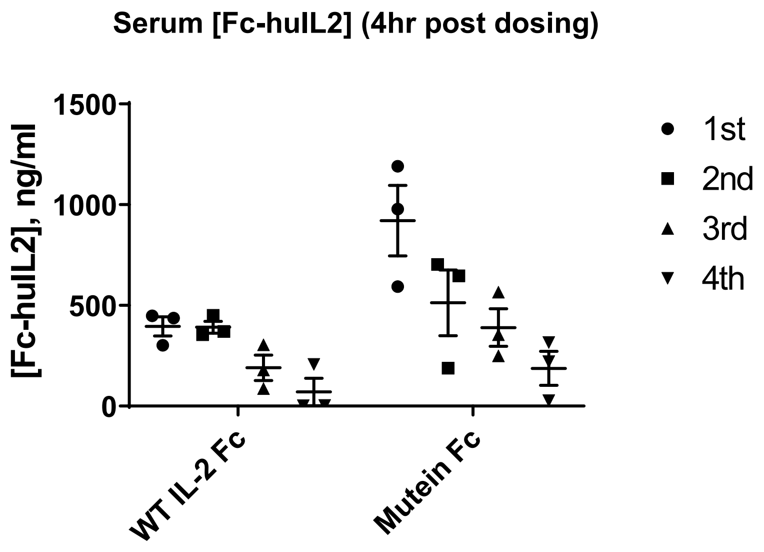

Fig. S6. Serum concentration of human IL-2 with repeat dosing in mice.
The concentration of huIL-2 in mouse serum was measured at 4 hours after each of the 4 weekly dosing of WT IL-2 or an attenuated mutein. *n=3* per group, shown are the mean of the group with error bars indicating SEM.

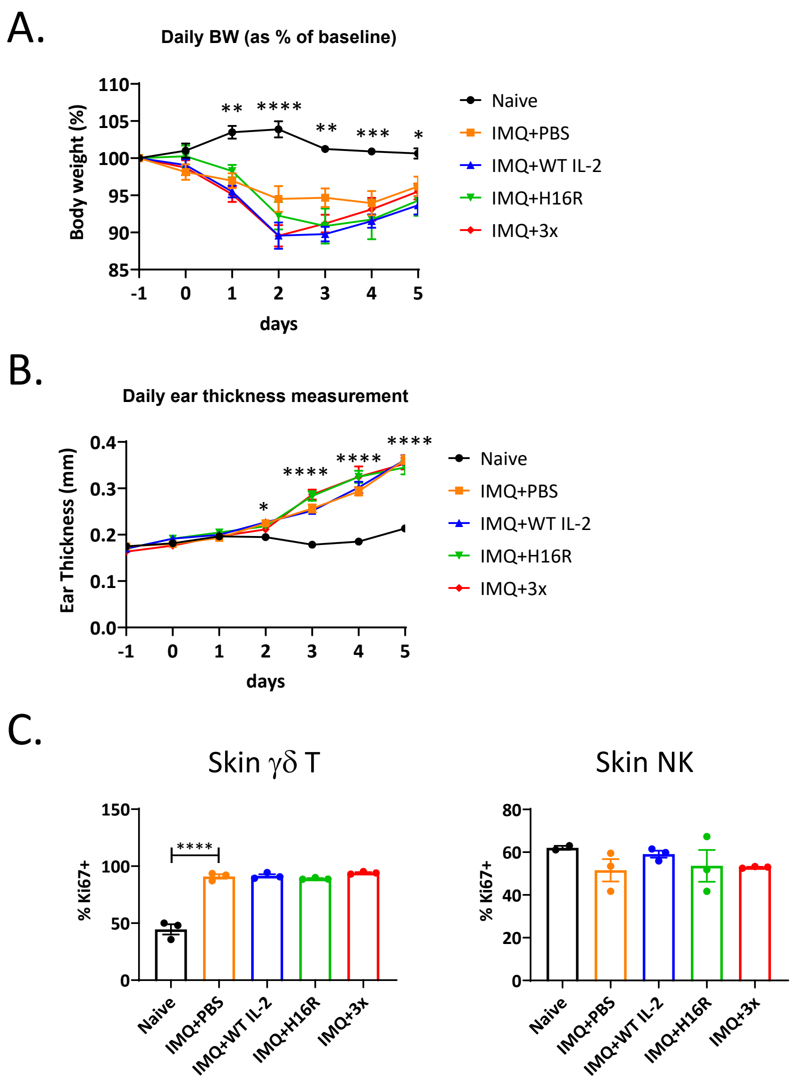

Fig. S7. Additional data from the IMQ psoriasis study.
Daily measurements of (A) body weight as percent of baseline on day -1 and (B) ear thickness in mm are shown for the duration of the study in the 5-day IMQ psoriasis model. IL-2 is administered by s.c. injection on day -1 at 25 μg per mouse and daily IMQ cream application began on day 0. Two-way ANOVA test, *p<0.05; **p<0.01; ***p<0.001;****p<0.0001, for Naïve vs IMQ+PBS. (C) Ki67 expression in skin γδ T and NK cells in naïve and diseased mice at study end with or without the IL-2 treatment. One-way ANOVA test, ****p < 0.0001. All graphs represent the mean values for the group, and the error bars indicate the SEM values. Shown are representative of two independent experiments.
